# *Neuron Navigator 1* (*Nav1*) regulates the response to cocaine in mice

**DOI:** 10.1038/s42003-023-05430-9

**Published:** 2023-10-18

**Authors:** Jared R. Bagley, Yalun Tan, Wan Zhu, Zhuanfen Cheng, Saori Takeda, Zhouqing Fang, Ahmed Arslan, Meiyue Wang, Yuan Guan, Lihua Jiang, Ruiqi Jian, Feng Gu, Isabel Parada, David Prince, J. David Jentsch, Gary Peltz

**Affiliations:** 1 Department of Psychology, Binghamton University, Binghamton, NY USA; 2 Department of Anesthesiology, Pain and Perioperative Medicine Stanford University Medical School, Stanford, CA USA; 3 Department of Genetics, Stanford University Medical School, Stanford, CA USA; 4 Department of Neurology, Stanford University Medical School, Stanford, CA USA; 5 Present Address: Department of Biological Sciences, University of North Texas, Denton, USA

**Keywords:** Genome-wide association studies, Addiction

## Abstract

Genetic variation accounts for much of the risk for developing a substance use disorder, but the underlying genetic factors and their genetic effector mechanisms are mostly unknown. Inbred mouse strains exhibit substantial and heritable differences in the extent of voluntary cocaine self-administration. Computational genetic analysis of cocaine self-administration data obtained from twenty-one inbred strains identified *Nav1*, a member of the neuron navigator family that regulates dendrite formation and axonal guidance, as a candidate gene. To test this genetic hypothesis, we generated and characterized *Nav1* knockout mice. Consistent with the genetic prediction, *Nav1* knockout mice exhibited increased voluntary cocaine intake and had increased motivation for cocaine consumption. Immunohistochemistry, electrophysiology, and transcriptomic studies were performed as a starting point for investigating the mechanism for the *Nav1* knockout effect. *Nav1* knockout mice had a reduced inhibitory synapse density in their cortex, increased excitatory synaptic transmission in their cortex and hippocampus, and increased excitatory neurons in a deep cortical layer. Collectively, our results indicate that *Nav1* regulates the response to cocaine, and we identified *Nav1* knockout induced changes in the excitatory and inhibitory synaptic balance in the cortex and hippocampus that could contribute to this effect.

## Abstract (web summary)

Mice lacking *neuron navigator 1* (*Nav1*) exhibit increased voluntary cocaine intake, potentially due to altered excitatory and inhibitory synaptic balance in the cortex and hippocampus.

## Introduction

While a substantial proportion of the risk for developing a SUD is genetic^1,2^, the genes and alleles that influence susceptibility to a SUD remain largely unknown. Just as in the human population, inbred mouse strains exhibit substantial and heritable differences in their responses to commonly abused drugs, which has enabled murine models for many human SUD behaviors to be generated^3^. Inbred mouse strains have been analyzed to identify genetic factors affecting addiction-related behaviors and their responses to drugs with high abuse potential^4–8^. While we do not expect that murine studies will be likely to identify the same genetic factors that are operative in humans, the neurobiological mechanisms that underlie SUD risk are likely to converge in mice and humans^3^. Hence, characterizing genetic factors that affect the responses of inbred mouse strains to these drugs could (i) increase our understanding of the neurobiological pathways that are impacted by them; (ii) reveal how addiction-related behaviors are generated; and (iii) could help to generate new approaches for preventing drug addiction in humans^9^. Among the various rodent assays, the cocaine self-administration assay (CSA) is considered a gold-standard assay for studying cocaine-related behavior, neurobiology, and genetics in rodent populations^10–12^. Mice are fitted with a jugular catheter and placed in an operant conditioning box where they actuate a lever to trigger cocaine infusions, which enables the extent of CSA to be measured. The rate of CSA reflects the reinforcing potential of cocaine^13^, and inter-strain differences in the magnitude of CSA reflect the propensity that a strain could misuse cocaine^14–16^.

We analyzed a murine genetic model for CSA and identified a candidate gene (*Nav1*) that could regulate cocaine responses. Analysis of *Nav1* KO mice confirmed that Nav1 had a strong effect on the level of voluntary cocaine consumption; and that it also impacted food reinforcement, which is another addiction-related trait. Some potential insight into the mechanism for this was provided by finding that the *Nav1* KO altered the excitatory/inhibitory synaptic balance in hippocampal and cortical brain regions.

## Results

### Identification of Nav1 as a candidate gene affecting CSA

**Fig. 1:**
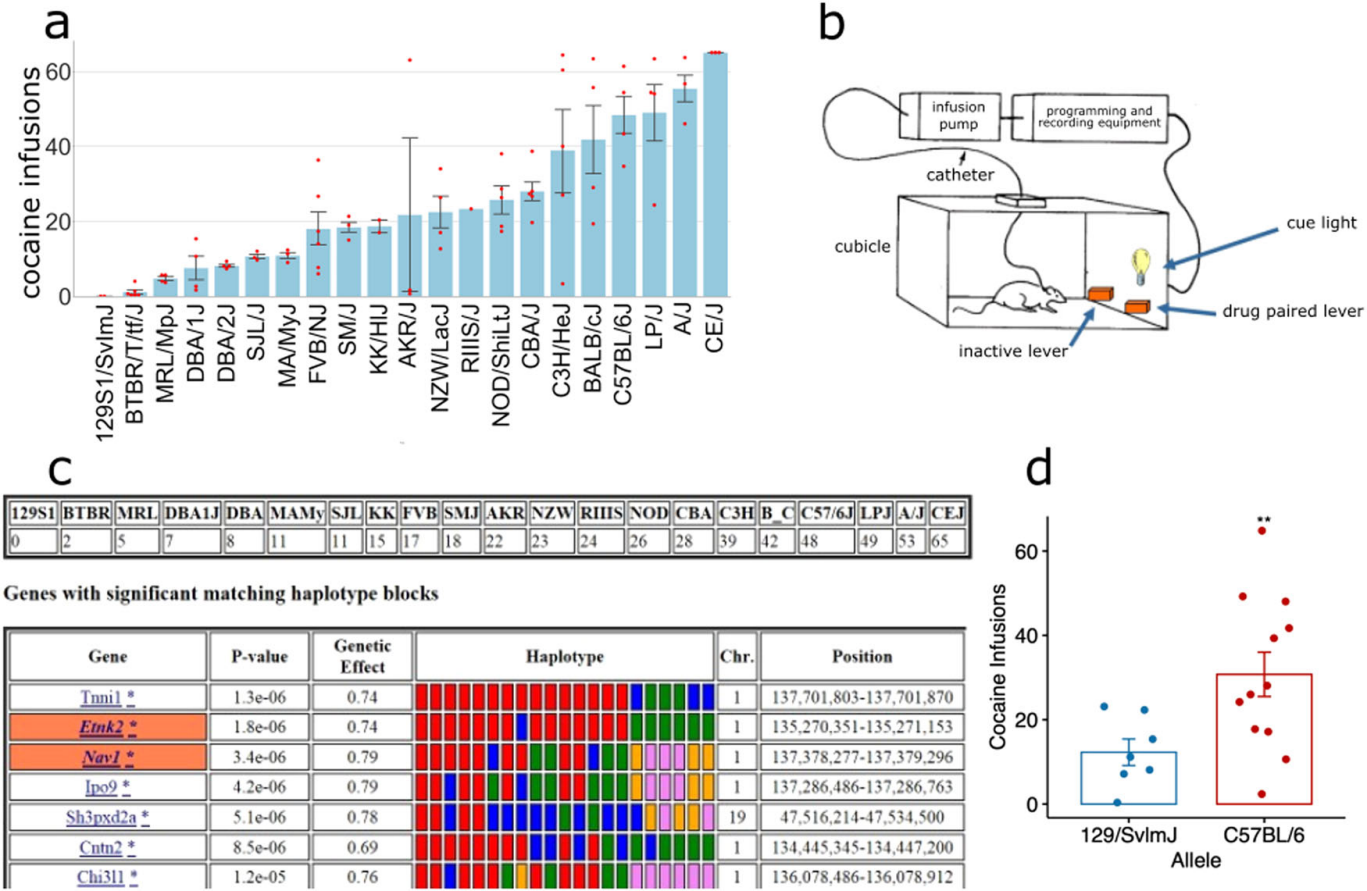
Identification of *Nav1* as a candidate gene effecting CSA. **a** The average number of cocaine infusions earned during the last 3 days of a 10-day CSA session was measured for each of 21 inbred strains. Each bar shows the strain mean ± SEM (*n* = 1–6 mice per strain). Scatter points represent infusions by individual mice. **b** A diagram of the CSA assay. **c** The HBCGM analysis output is shown. The top panel shows the average number of cocaine infusions for each strain, and the bottom panel shows the top 7 genes identified by HBCGM analysis of this data. The gene symbol, genetic effect size, chromosomal position, and the *p* values for the genetic association are shown. If the box surrounding the gene symbol has an orange color, it indicates that the haplotype block has a SNP allele causes a significant change in the protein sequence. In the haplotype box, the haplotypic pattern is shown as colored rectangles that are arranged in the same order as the input data shown above; strains with the same-colored rectangle have the same haplotype. **d** The mean ± SEM for the number of cocaine infusions (*y*-axis) self-administered by strains with different *D198E* cSNP alleles in *Nav1* are shown. There is a statistically significant increase (*p* = 0.007) in number of cocaine infusions self-administered by the 12 strains (C57BL/6, CE/J, LP/J, Balbc/J, C3H/HeJ, CBA/J/ NOD/J, RIIIS, SMJ, FVB, MaMy, BTBR) with the C57BL/6J allele (*D198) (*average of 32.5 ± 5.2 infusions) relative to the 8 strains (NZW, AKR, KK, SJL, DBA2, DBA1, MRL, 129Sv1) with the 129Sv1 allele (*E198)* (average of 11.3 ± 2.8 infusions).

Voluntary CSA was examined over a 10-day period (0.5 mg/kg/infusion) in adult male mice of 21 inbred mouse strains. During the last 3 days of testing, the strains exhibited very different levels of CSA (range 0–65 infusions), and the heritability for the inter-strain differences in this measurement was 0.68 (Fig. 1a). HBCGM analysis of the CSA data for the 21 inbred strains revealed that the allelic patterns within multiple genes that were co-localized within a region on chromosome 1 (134–137 MB) were most strongly associated with the inter-strain differences in CSA (Fig. 1). Among the genes within the chromosome 1 region (134–137 MB) with allelic patterns that correlated with inter-strain differences in CSA, only two (*Nav1, Etnk2*) had SNP alleles that altered the predicted amino acid sequence. However, *Nav1* was the only one of these genes with a high level of mRNA expression in key brain regions, and its allelic pattern was not associated with population structure^17^ (Supplementary Table 2). There were 2163 SNPs within or near (±10 kB) the *Nav1* gene, which included 20 synonymous SNPs, and 3 cSNPs (*D198E, P1366L, A911V)* that altered the Nav1 amino acid sequence (Supplementary Table 3). Also, two SNPs (*D198E, A911V)* were within predicted sumoylation (193–202 -EAAVS**D**DGKS) and phosphorylation (910–917 -TAPSEEDT) motifs. While we certainly do not know the specific *Nav1* alleles that contribute to the different levels of CSA exhibited by the inbred strains, it is noteworthy that the level of CSA was correlated with the *Nav1 D198E* allele of an inbred strain (*p* = 0.007) (Fig. 1d). Several other factors suggested that genetic variation within *Nav1* could affect CSA. (i) *Nav1* is expressed predominantly in the nervous system^18^. (ii) Nav1 plays a role in neuronal development and in the directional migration of neurons^19^ by regulating neurite outgrowth through effects on cytoskeletal remodeling^20^, which are processes that have been associated with addiction-induced changes in brain^21^. (iii) Multiple *Nav1* mRNA isoforms are produced by alternative splicing^18^, which provides another mechanism by which allelic differences could impact a phenotype. (iv) A prior HBCGM analysis led to the discovery that alleles within another axonal guidance protein (*Netrin 1*) affected multiple maladaptive responses to opioids^7^. (v) Analysis of striatal *Nav1* mRNA expression levels in a BXD recombinant inbred strain panel indicated that *cis* acting alleles regulate its expression (Supplementary Fig. 1). (vi) When the *Nav1* mRNA expression QTL was evaluated for correlation with the entire database of phenotypes measured in this panel of recombinant inbred strains, the highest correlation was an inverse one with striatal *dopamine receptor D2 (Drd2)* mRNA levels. This correlation is specific in that striatal *Drd1* mRNA expression is positively associated with *Nav1* mRNA levels (Supplementary Fig. 1), and this is noteworthy since low Drd2 expression and function is a biomarker for addiction vulnerability in animals and humans^22–26^. All of these features suggest that *Nav1* allelic variation could impact Drd2 dopamine-dependent signaling within the striatum, and thus, responses to potentially addictive drugs.

### Generation and characterization of a Nav1 knockout (KO) mouse

Due to its large size, the presence of many SNPs within *Nav1*, and the multiple mechanisms by which *Nav1* alleles could impact a genetic trait, the genetic hypothesis was tested by producing a homozygous *Nav1* KO mouse on a strain background (C57BL/6J) that exhibited a moderate level of CSA^27^ (Supplementary Fig. 2). Single molecule FISH analysis indicated that full length *Nav1* mRNA is expressed in C57BL/6J (but not in *Nav1* KO) brain tissue, while the *Nav1* KO transcript is exclusively expressed in *Nav1* KO mice (Supplementary Fig. 3, Supplementary Table 5). Proteomic analysis confirmed that Nav1 protein was absent in *Nav1* KO brain tissue (see Methods). MRI brain scans revealed that *Nav1* KO mice did not have gross neuroanatomical abnormalities. *Nav1* KO mice had a slightly smaller overall brain volume, which was consistent with their smaller body size (vs. C57BL/6J mice). After normalization of hippocampal volume to overall brain size, there was no significant difference in their relative hippocampal volume (vs. C57BL/6J mice) (Supplementary Fig. 4).

### The Nav1 KO effect on voluntary CSA

**Fig. 2:**
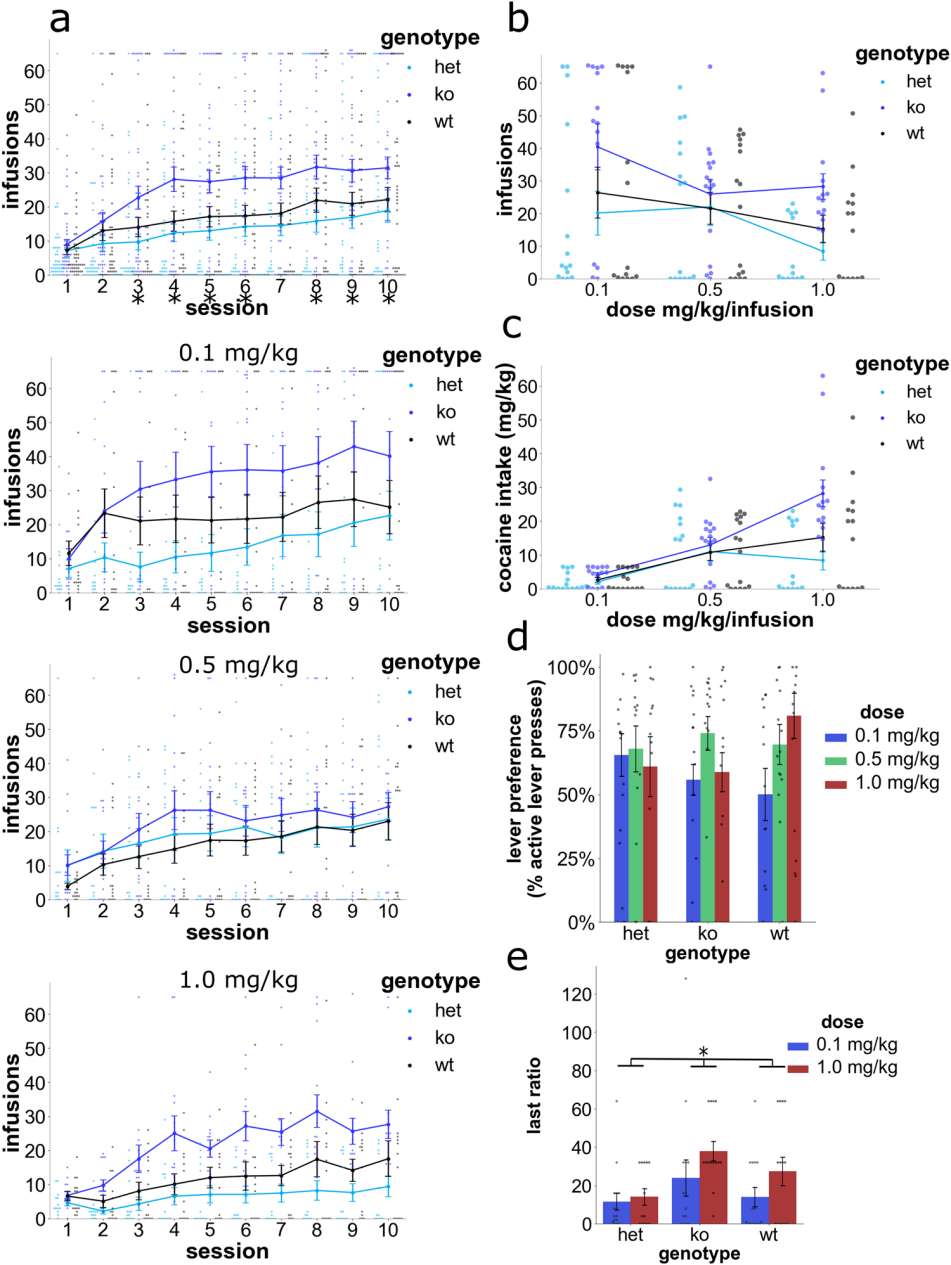
CSA dose responses for wildtype, HET, and homozygous *Nav1* KO mice. All graphs include the mean ± SEM. **a** Infusions earned in 10 sessions of CSA across genotype and across/within dose (*n* = 13–16 mice per genotype/dose). *Nav1* KO mice self-administered more cocaine than wildtype and HET mice from session 3 on (upper graph represents genotype means across dose; *p < 0.05 between KO and wildtype/HET for post hoc comparisons across dose and within session). Lower graphs visualize responding within each dose (however no genotype-by-dose interaction was detected). **b** A visualization of the dose response curve for the number of cocaine infusions during last 3 sessions, which are averaged for each dose for mice of the indicated genotype. *Nav1* KO mice displayed an upward shift in the dose response curve. **c** Visualization of the dose response curve for cocaine intake (mg/kg) during the last 3 sessions for each cocaine dose. Similar to the number of infusions, *Nav1* KO mice displayed an upward shift in the dose-response curve for cocaine intake. **d** Active lever preference (i.e., the % of correct lever presses) during the last 3 sessions are shown for each of the 3 doses of cocaine. There was no difference in active lever presses among the groups of mice with different genotypes. **e** CSA was m**e**asured at two different doses (0.1 and 1.0 mg/kg) in Wt, Het and *Nav1* KO mice (*n* = 13–16 mice per group) using a progressive ratio test, where the number of active lever presses required for receiving the next cocaine infusion is progressively doubled from that required for the previous infusion. The number of lever presses required to earn the last cocaine infusion (i.e., the last ratio) provides an assessment of their motivation for cocaine consumption. *Nav1* KO mice achieved a significantly greater last ratio than wildtype or Het mice (*p < 0.05; main effect of genotype).

A between-subjects dose-response design was used to compare the level of CSA between wildtype, heterozygous *Nav1* KO (HET) and homozygous *Nav1* KO mice. The data were analyzed by mixed ANOVA with session as a repeated measure and genotype/dose/sex as between subjects factors. Analysis of this data indicated that there was a clear effect of genotype [F[2,113) = 8.5, *p* < 0.001], and sex [F[1,113) = 7.2, *p* = 0.008] on the number of cocaine infusions earned. A session-genotype interaction [F[8.6,483.3) = 2.8, *p* = 0.003], along with a main effect of session [F[4.3, 483.3) = 4.4, *p* = 0.001] were also found. Post hoc, pairwise comparisons indicated that the *Nav1* KO mice earned more infusions than HET *Nav1* KO mice (*p* < 0.05) following session 2 and more infusions than wildtype mice following session 3 (except for session 7 (*p* = 0.052)). These results indicate that *Nav1* KO mice self-administered more cocaine infusions and this effect was not impacted by dose. Furthermore, their increased level of consumption appeared during the early period of cocaine IVSA acquisition and was maintained throughout the 10 days of testing (Fig. 2a, b). The sex effect indicated that males consumed more cocaine than females across all doses and genotypes tested (Supplementary Fig. 5).

Cocaine intake was analyzed by ANOVA with session as a repeated measure and genotype/dose/sex as between subject factors. This analysis indicated that *Nav1* KO mice self-administered a larger amount of cocaine than HET and wildtype mice at all doses (session-genotype interaction [F(7.7,434.5) = 3.0, *p* = 0.003], and that there was a main effect of session [F(3.8,434.5) = 7.5, *p* < 0.001], and genotype [F(2,113) = 9.5, *p* < 0.001]). *Nav1* KO mice self-administered more cocaine than HET mice after session 2 and more than wild-type mice after session 3 (*p* < 0.05). Mice of all three genotypes self-administered more cocaine as the dose increased (i.e. there was a main effect of dose [F(2,113) = 10.3, *p* < 0.001]) (Fig. 2c). Also, a genotype-sex interaction [F(2,113) = 3.5, *p* = 0.034] suggested that the sex differences were dependent on genotype. A post-hoc analysis indicated that there were significant sex differences (males > females) in wildtype (*p* < 0.001) but not in *Nav1* KO (*p* = 0.912) or HET (*p* = 0.218) mice. Similarly, when the preference for the active lever was assessed during the last 3 days of CSA, a genotype-sex interaction was detected [F(2,99) = 3.3, *p* = 0.041], and wildtype female mice had a lower preference than males (*p* = 0.017) (Supplementary Fig. 5). We found no significant differences in lever preference between the genotypes (Fig. 2d).

We then assessed their motivation for self-administering cocaine using a progressive ratio schedule of reinforcement (0.1 and 1.0 mg/kg doses), where the number of active lever presses required for receiving the next cocaine infusion was progressively doubled from the response requirement of the previous infusion. Analysis of the last ratio of lever presses required to earn a cocaine infusion indicated that there was a genotype-dose-sex [F(2,73) = 3.8, *p* = 0.029] and genotype-sex [F(2,73) = 8.3, *p* = 0.001] interaction, and a main effect of genotype [F(2,73) = 3.9, *p* = 0.025]. Analysis within sex revealed that there was a genotype-dose interaction in males [F(2,38) = 4.7, *p* = 0.015] but not in females [F(2,35) = 1.3, *p* = 0.292]. Males of the wildtype genotype earned a higher last ratio under the 1 mg/kg dose relative to the 0.1 mg/kg dose (*p* = 0.043) (Supplementary Fig. 5). Overall, these data indicate that *Nav1* KO mice maintained their greater level of cocaine intake under a progressive ratio schedule (Fig. 2e). In contrast, the acute locomotor responses of *Nav1* KO mice after experimentally administered cocaine were not different from those of C57BL/6J mice (Supplementary Fig. 6).

### The Nav1 KO also impacts food self-administration (FSA)

**Fig. 3:**
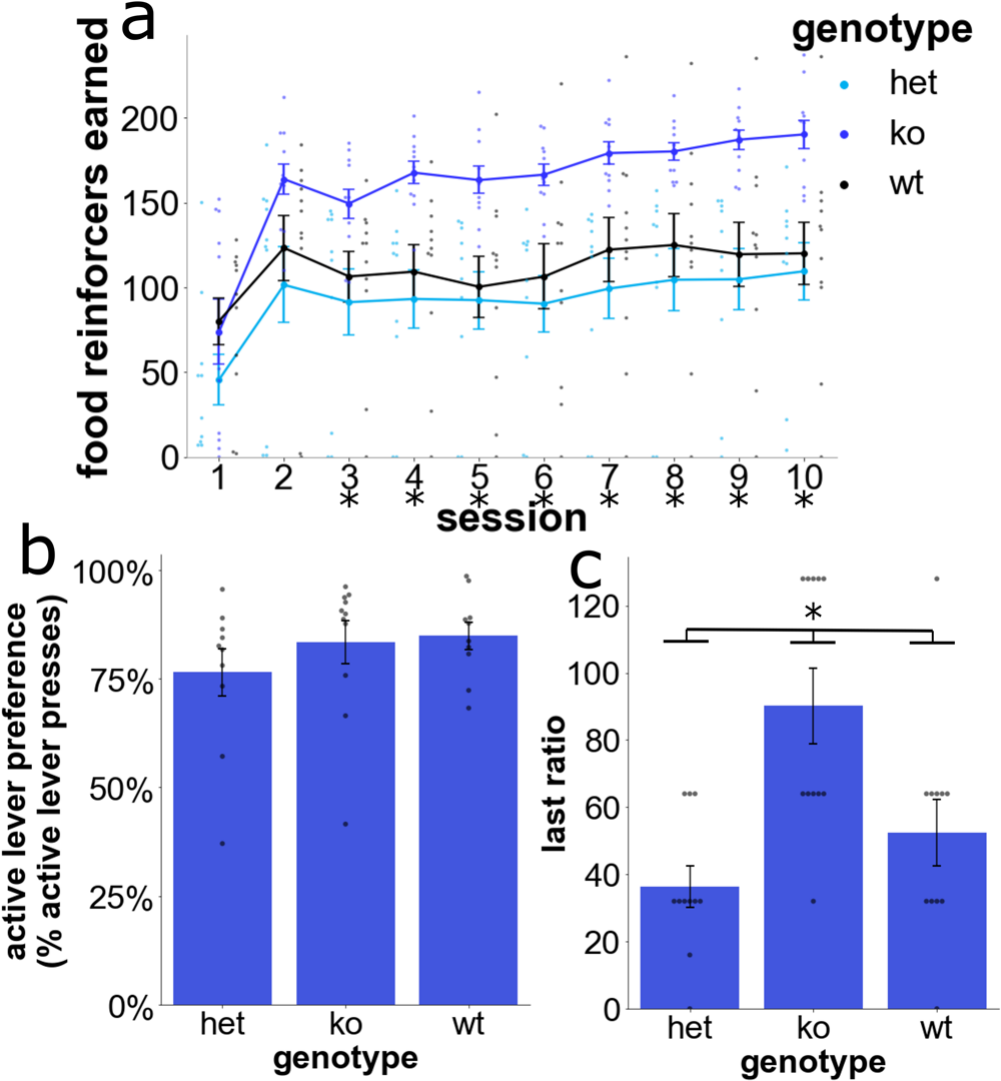
Food self-administration (FSA) by wildtype, HET, and *Nav1* KO mice (*n* = 11 mice per group) was measured in 10 daily sessions. All graphs include the mean ± SEM, and the response of an individual mouse is shown as a dot. **a** *Nav1* KO mice had a continuously higher level of FSA than wildtype or HET mice beginning from session 4 (*p < 0.05). **b** The percentage of active lever presses (Active Lever Preference) was measured during the last 3 sessions. No differences in active lever preference was detected between mice with the three different genotypes. **c** FSA was measured in male and female Wt, Het, and *Nav1* KO mice (*n* = 5–6 mice of each sex per group) using a progressive ratio test where the number of active lever presses required for receiving the next food reinforcer is progressively doubled from that required for the previous one. The number of lever presses required to earn the last food reinforcer (i.e., the last ratio) provides an assessment of their motivation for food consumption. Male and female *Nav1* KO mice achieved a significantly greater last ratio than Het or Wt mice (*p* < 0.5).

To determine whether the *Nav1* KO impacted self-administration of a non-drug reinforcer, mice were tested using a FSA procedure that was similar to that of CSA testing except that a chocolate solution was delivered as the reinforcer. Analysis of the number of food reinforcers earned indicated that there was a genotype-session interaction [F(5.1,66.3) = 2.5, *p* = 0.036] and a main effect of genotype [F(2,26) = 6.5, *p* = 0.005] and session [F(2.6,234) = 26.2, *p* < 0.001] (Fig. 3a). The pairwise comparisons, which examined the effect of different genotypes on the FSA results within each session, revealed that *Nav1* KO mice earned a greater number of food reinforcers than HET (from session 3 on) or wildtype (from session 4 on except for session 8) (*p* = 0.059) mice (Fig. 3a). Assessment of active lever preference over the last 3 sessions did not indicate any significant genotypic effect (Fig. 3a). We observed that KO mice weighed less at baseline (bodyweight mean ± SD, KO male 26.6 ± 1.2, female 19.3 ± 1.1; HET male 27.4 ± 1.6, female 23.0 ± 0.8; wildtype male 28.5 ± 0.3, female 22.9 ± 1.2). *Nav1* KO mice also lost more weight than HET or wildtype mice during the period of food restriction, which may be due to their baseline increase in locomotor activity (percent of baseline bodyweight after food restriction (mean ± SD), KO 86% ± 6%; HET 94% ± 2%; wildtype 92% ± 2%). Therefore, the FSA test was repeated without food restriction. These results confirmed that *Nav1* KO mice earned more food reinforcers: there was a session-genotype interaction [F(5.5,35.6) = 3.0, *p* = 0.02], and there was a main effect of genotype [F(2,13) = 4.0, *p* = 0.045]) on the FSA results. These results indicate that *Nav1* KO effect on FSA was not due to food-deprivation. After 10 sessions on a fixed ratio1 (FR1) schedule, the mice underwent a progressive ratio test. Analysis of the last ratio achieved also indicated that there was a main effect of genotype [F(2,27) = 8.1, *p* = 0.002]. Post hoc, pairwise comparisons indicated that *Nav1* KO mice achieved a greater last ratio than wildtype (*p* = 0.026) and Het (*p* = 0.001) mice (Fig. 3c). Taken together, the FSA results indicate that the *Nav1* KO also altered food reinforcement.

### Nav1 KO mice have alterations in learning, memory, and in other behaviors

Since it was postulated that addictive drugs coopt the reward pathways used for learning and memory^28^, we investigated whether learning and memory were impacted by the *Nav1* KO. To do this, we first assessed whether the ability to recognize a novel object was altered in *Nav1* KO mice. While C57BL/6J mice spent significantly more time exploring a novel object relative to a familiar one, homozygous and HET *Nav1* KO mice spent a similar amount of time exploring the two objects (Supplementary Fig. 7a) (novelty: *p* = 0.0008; genotype × novelty: *p* = 0.45). There were significant differences in the time that C57BL/6J mice spent with novel vs familiar objects (*p* = 0.045), but this difference was not manifested by HET (*p* = 1.0) or homozygous *Nav1* KO (*p*-value = 0.87) mice. When spatial learning and memory capabilities were assessed using the Barnes maze test, *Nav1* KO mice had a significantly reduced ability to correctly recognize the escape hole relative to HET (*p* < 0.01) or C57BL/6J (*p* < 0.0001) mice (Supplementary Fig. 7b). There were no significant differences in the number of primary errors made (*p* = 0.36) or in the total distance traveled (*p* = 0.16) between the three groups of mice. We next investigated whether the *Nav1* KO impacted anxiety or locomotor activity. *Nav1 KO* mice exhibited altered exploratory behavior in the elevated plus maze; they spent more time in the open arms relative to HET (*p* = 0.005) or C57BL/6J mice (*p* = 0.006) (Supplementary Fig. 8a). Open field testing revealed that *Nav1* KO mice had a significant increase in the total distance traveled relative to HET mice (*p* = 0.01); and spent a decreased percentage of time in the open field relative to C57BL/6J mice (*p* = 0.04) (Supplementary Fig. 8b). However, *Nav1* KO mice had normal motor coordination in the rotarod test (Supplementary Fig. 8c). These results indicate that *Nav1* plays an important role in learning and memory capabilities, and that it also effects the level of anxiety and exploratory behavior.

### Increased excitatory hippocampal synaptic transmission in Nav1 KO mice

Since Nav1 is involved in neuronal development and migration^19,20^, we quantitatively examined the impact of the *Nav1* KO on synapse formation. *Nav1* KO mice had a significant increase in the number of excitatory synapses in the dentate gyrus of the hippocampus (vs C57BL/6J mice, *p* = 0.03), but inhibitory synapse density was not changed (*p* > 0.99) (Supplementary Fig. 9a). In vitro electrophysiological recordings from granule cells in the dentate gyrus of the hippocampus showed a significant increase in the frequency (but not in the amplitude) of miniature excitatory postsynaptic currents (mEPSCs) in *Nav1* KO vs C57BL/6J mice (Supplementary Fig. 9a), which indicates that there is increased excitatory synaptic transmission in the dentate gyrus of *Nav1* KO mice. In contrast, there was no significant difference in the frequency or amplitude of the miniature inhibitory postsynaptic currents (mIPSCs) measured in *Nav1* KO vs C57BL/6J mice. There was also a significant decrease in the number of inhibitory synapses formed in the prefrontal cortex (PFC) of *Nav1* KO mice (vs. C57BL/6J, *p* = 0.008), while excitatory synapse density was not altered (Supplementary Fig. 10). Since the nucleus accumbens (NAc) is a critical center for responses to addictive drugs^29^, we compared the cells in the NAc of *Nav1* KO and C57BL/6J mice by immunohistochemical staining. There was a similar density of neuronal cells in the NAc of *Nav1* KO and C57BL/6J mice, as shown by the equal number of neuronal nuclei marker^+^ and neurofilament H^+^ cells. However, glial fibrillary acidic protein (GFAP) staining indicated that there was a slight trend toward an increase in the number of glial cells in the NAc of *Nav1* KO mice (Supplementary Fig. 10). Thus, *Nav1* KO mice have an increase in hippocampal excitatory synapses and synaptic transmission, and a decrease in the number of inhibitory synapses formed in the PFC.

### Nav1 KO-induced transcriptomic changes

**Fig. 4:**
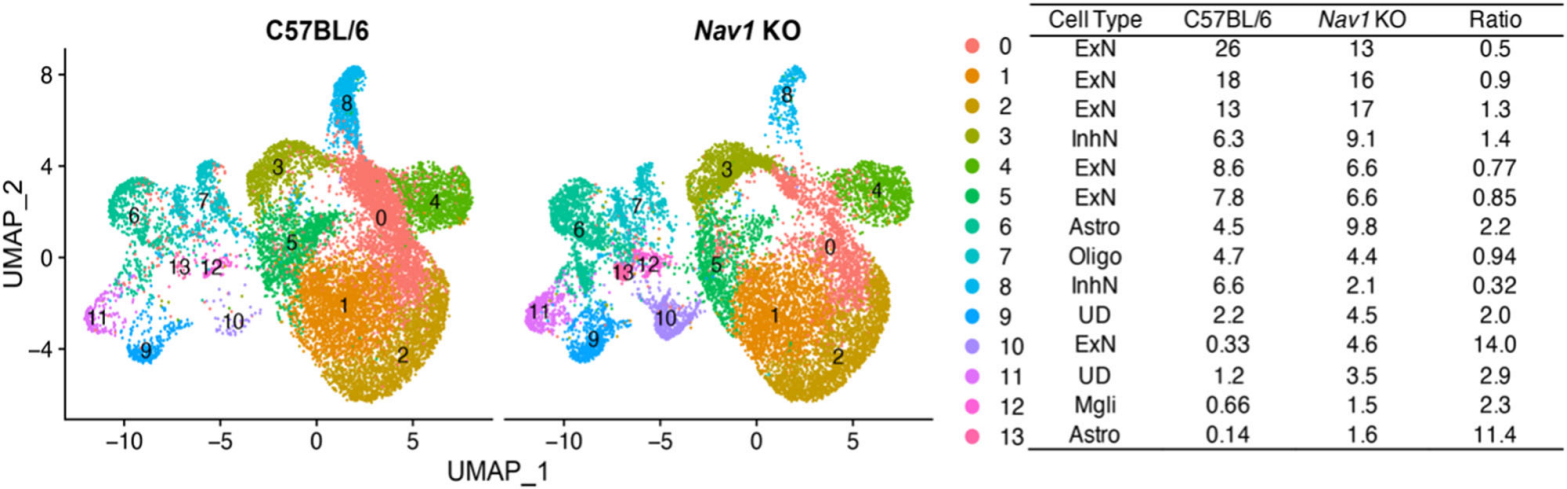
snRNA-Seq analysis of *Nav1* KO and isogenic C57BL/6J PFC tissue. UMAP plots show the data for the C57BL/6J and *Nav1* KO cells. Each dot represents an individual cell. The table indicates the dot color for each cluster, the percentage of cells in each cluster, and the ratio of the cell percentages (*Nav1 KO*/C57BL/6J) in C57BL/6J and *Nav1* KO PFC. This dataset analyzed the expression of 21,892 genes in 28,686 nuclei. Based upon total transcriptomic differences, the PFC cells were separated into 14 different clusters. Based upon canonical marker expression, the clusters are derived from the five lineages: astrocytes (6, 11), microglia (12), oligodendrocytes (7); and inhibitory (3, 8) and excitatory (0, 1, 2, 4, 5, 10) neurons. The lineages for two clusters (9, 11) were undefined (UD) because they expressed markers from different cell lineages. Of particular interest, the number of cells in an inhibitory neuron cluster (cluster 8) is three-fold decreased in *Nav1* KO mice, and there is a 14-fold increase in the number of cells in an excitatory neuron cluster (cluster 10) in *Nav1* KO mice.

The PFC regulates higher cognitive functions (i.e., learning, memory, and decision making) and forms connections with multiple other brain regions^30,31^, and we identified synaptic changes in the PFC of *Nav1* KO mice. Therefore, we examined *Nav1* KO-induced transcriptomic changes in the PFC. snRNA-Seq was performed on 28,686 high quality PFC cells obtained from *Nav1* KO mice *(*14,056 cells; 68,637 reads per cell) and from age-matched, isogenic C57BL/6J mice (14,630 cells, 56,980 reads per cell with a mean of 1438 genes per cell). The PFC cells were separated into 14 clusters, which based upon their pattern of marker mRNA expression^32^, were derived from 4 lineages: neurons, astrocytes, oligodendrocytes, and microglia (Fig. 4, Supplementary Figs. 11–13). The eight neuronal clusters were separated into six excitatory (0–2, 4, 5, and 10) and two (3, 8) inhibitory neuronal clusters. Consistent with prior findings^32^, excitatory neurons were 5 to 7-fold more abundant than inhibitory neurons (52–75% vs 7–14% of the total). The cell clusters and their distribution in our C57BL/6J PFC data were very similar to that of a prior analysis^32^ (Supplementary Fig. 14). Clusters 10 and 5 had the highest levels of *Nav1* mRNA (Supplementary Fig. 11). Cluster 10 was of particular interest since its abundance was most increased (14-fold) in *Nav1* KO (vs C57BL/6J) mice. Cluster 10 uniquely expressed an excitatory neuronal marker (*Tshz2*) that is characteristic of cortical layer 5 (L5) cells (Supplementary Fig. 11), which form projections to extra-cortical brain regions and are in the cortical layer whose transcriptome was most altered during cocaine withdrawal^32^. Pathway enrichment analysis of the differentially expressed genes (DEGs) in cluster 10, revealed that many of the enriched pathways were associated with neuronal guidance, synapse or signaling functions; and 7 DEGs (*Gria3, Grin2b, Grin2a, Camk2a, Ppp3ca, Gria4, Prkcb*) were associated with an amphetamine addiction pathway (*p* = 0.000025) (Supplementary Fig. 15). Clusters 5 and 10 also had the highest levels of expression of two mRNAs (*Rab3a*^33^, *Cck*^34^), whose expression levels were altered during cocaine withdrawal^32^ (Supplementary Fig. 16). Cluster 5 uniquely expressed a transcription factor (*Etv1*, aka *Er8*) that is required for generating habitual behaviors^35^. The inhibitory neurons in cluster 8 were of interest because their abundance was 3-fold decreased in the PFC of *Nav1* KO mice, and they uniquely expressed *Drd1* mRNA (Supplementary Fig. 12). In contrast, *Drd2* mRNA was not expressed at detectable levels in any cluster. Of importance, the mRNA levels measured in PFC obtained from C57BL/6J and *Nav1* KO mice are not necessarily related to the *Drd1-2* mRNA levels in measured in the striatum of the recombinant inbred strains. In summary, the transcriptomic analysis indicates that the *Nav1* KO affected neurons located in a deep cortical layer, and some of these cells had transcriptomic changes that were similar to those associated with cocaine withdrawal.

## Discussion

Our analysis of a murine genetic model identified *Nav1* as a candidate gene that influences voluntary cocaine consumption, and this genetic finding was confirmed by the increased level of CSA exhibited by *Nav1* KO mice across a broad range of cocaine doses. The effect of the *Nav1* KO on voluntary cocaine consumption occurred early; it was maintained throughout a 10-day period of CSA testing. Moreover, subsequent testing revealed that *Nav1* KO mice exhibited an increased motivation for consuming cocaine^36^. Since *Nav1* KO and wildtype mice did not exhibit differences in lever preference or in cocaine-induced acute locomotor responses; neither indiscriminate lever pressing nor differential locomotor effects could be responsible for their increased cocaine-intake. Although HET mice did not significantly differ from wildtype mice, a potential trend towards lower cocaine intake in HET mice is present in the data, suggesting that the heterozygous allele state alters cocaine intake in the opposite direction of the homozygous knockout state. Overall, these results indicate that the reinforcer efficacy of cocaine is increased in *Nav1* KO mice. The *Nav1* KO had a similar effect on food reinforcement, which suggests that the effect of the *Nav1* KO could be generalized across drug and non-drug reinforcers. It has been postulated that a common neural circuitry may underlie food and drug addictions^37^, and genetic variation in *Cyfip1* had effects on both cocaine and food reward^5,38^. *Nav1* KO mice also exhibited reduced anxiety-like behavior. An inverse relationship between anxiety-like behavior and CSA^39^ and ethanol drinking^40^ was previously observed in rats. While the *Nav1* results also indicate that the neural circuits mediating drug reward, food reward and anxiety may have some overlap, additional research is necessary to further explore this possibility.

After finding that the *Nav1* KO affected CSA and FSA, we also found that *Nav1* KO mice also exhibited reduced spatial learning and memory. Therefore, we performed a series of studies to identify potential mechanism(s) for these *Nav1* KO-effects. (i) Brain MRI demonstrated that the *Nav1* KO did not cause gross structural changes. (ii) Since Nav1 is involved in neuronal development and migration, we examined the *Nav1* KO effect on neurons and synapses. *Nav1* KO mice had a significant increase in excitatory synapse density in the dentate gyrus and a significant decrease in inhibitory synapse density in the PFC. Consistent with these results, EPS studies revealed that cells in the dentate gyrus of *Nav1* KO mice had an increased frequency of mEPSCs. (iii) The PFC was analyzed by scRNA-Seq because it regulates higher cognitive functions that were impacted by the *Nav1* KO (i.e., learning and memory) and because of the observed change in excitatory and inhibitory synapse density in the *Nav1* KO PFC. The *Nav1* KO-induced alterations in the balance between excitatory and inhibitory synapses in the cortex and hippocampus provides a potential mechanism by which *Nav1* could impact learning, memory, and possibly the response to an addictive drug. This mechanism is consistent with the known role of *Nav1* in regulating neuronal development, directional migration^19^, and neurite outgrowth^20^. While various cortical and subcortical brain regions affect the response to addictive drugs, the PFC integrates reward-seeking and decision-making, through the inhibitory control it exerts^14,41^ through the dense reciprocal connections it forms with virtually all neuromodulatory centers within cortical and subcortical regions^42^. For example, PFC glutamatergic neurons connect to the NAc core, which can provide a top-down control mechanism for preventing food addiction behaviors^43^. Disruptions to hippocampal synaptic balance could cause the learning/memory deficits observed in *Nav1* KO mice, since these tests (particularly the Barnes maze) are hippocampal-dependent^44,45^. Since the hippocampus also has a role in cocaine responses^46^, *Nav1-*induced disruptions of hippocampal function could also affect CSA.

In summary, we demonstrated that *Nav1* plays a role in in addiction-related traits that include voluntary cocaine consumption and food reinforcement. Moreover, Nav1 also altered anxiety-like behavior, learning/memory, and the excitatory/inhibitory synaptic balance in hippocampal and PFC brain regions. However, further characterization of neural adaptations occurring in *Nav1* KO mice could enable the role of Nav1 and the pathways mediating addiction-associated behaviors to be more fully characterized. Moreover, generation and characterization of mice with brain a region-specific conditional *Nav1* knockout or of knockin mice with alterations in specific *Nav1* alleles are also needed to improve our understanding of the role that Nav1 plays in SUDs.

## Methods

### Mouse strains

All mouse procedures were approved by the Institutional Animal Care and Use Committees at Binghamton or Stanford University; and were conducted in accordance with the National Institute of Health Guide for Care and Use of Laboratory Animals, Eighth Edition. All mice were originally obtained from Jackson Laboratories, and the results are reported according to the ARRIVE guidelines^47^. The strains utilized for genetic mapping were bred for 7 generations or less prior to testing. *Nav1* KO mice (on C57BL/6J background) were maintained at Binghamton and at the Stanford University School of Medicine. Mice were housed in their home cage and maintained on *ad libitum* mouse chow (5L0D, Purina Lab Diet) and water. Mice were individually housed in polycarbonate cages (30 × 8 cm) with wood-chip bedding (SANI-CHIPS), a paper nestlet and a red polycarbonate hut.

### Cocaine self-administration (CSA)

Chronic indwelling jugular catheters were inserted and managed as described^27^. Twenty-one strains (*n* = 1–6 mice per strain, male mice, age range: 12–18 weeks) were tested for CSA acquisition in 10 consecutive daily sessions (Fixed-ratio-1 schedule of reinforcement, 0 or 0.5 mg/kg body weight of cocaine per infusion) that ran until 65 infusions were earned or 2 h passed, whichever came first. Cocaine hydrochloride (Sigma Aldrich; St Louis MO) was dissolved in sterile saline at a concentration of 0.17, 0.84, or 1.68 mg/mL to produce a freebase dose of 0.1, 0.5, or 1.0 mg/kg/infusion (infusion volume was 0.67 mL/kg/infusion). Testing occurred at the same time each day, during the light phase of a 12/12 h cycle. The animals were tested in Med Associates mouse self-administration chambers (55.69 × 38.1 × 35.56 cm, MED-307W-CT-D1, Med Associates, VT) that were fitted with 2 retractable ultrasensitive levers and that were housed within sound-attenuating cubicles. Assignment of the active infusion lever (right or left side of the box) was counterbalanced across strains/sex. Assignment of testing chamber minimized testing multiple mice from a given strain in the same chamber. Test sessions began with the activation of the white noise and the illumination of 5 stimulus lights on the back wall of the chamber. No priming infusion(s) were delivered. When a subject actuated the active lever, an infusion was delivered, the house light flashed, and the aperture lights turned off for 20 s. During this time-out period, contacts on the active lever were recorded but had no programmed consequence. Actuation of the inactive lever had no programmed consequence. The number of infusions earned, cocaine intake (mg/kg) (number of infusions X dose) and active lever preference (active lever presses/total lever presses) were key variables of interest. Active lever preference is incalculable if the mouse fails to press either lever, and consequently analysis of this variable by session leads to excessive missing data points. Therefore, the last 3 CSA sessions were collapsed by calculating preference across these days, within individual mice.

### Haplotype based computational genetic mapping (HBCGM)

The SNP database was generated by analysis of the genomic sequences of 47 classical (C57BL/6J, 129P2, 129S1, 129S5, AKR, A_J, B10, BPL, BPN, BTBR, BUB, BALB, C3H, C57BL10J, C57BL6NJ, C57BRcd, C57LJ, C58, CBA, CEJ, DBA, DBA1J, FVB, ILNJ, KK, LGJ, LPJ, MAMy, NOD, NON, NOR, NUJ, NZB, NZO, NZW, PJ, PLJ, RFJ, RHJ, RIIIS, SEA, SJL, SMJ, ST, SWR, TALLYHO, RBF, MRL) and 6 wild-derived (CAST, MOLF, PWD, PWK, SPRET, WSB) inbred strains as described in^48–50^. HBCGM was performed as described^51^ using modifications described in^52^. The chromosomal location and potential codon-changes for a SNP are annotated using predictive gene models from Ensembl version 65. The methods for calculation of the genetic effect size (η^2^) and for other mapping methods are provided elsewhere^53,54^.

### Generation and characterization of Nav1 KO mice at the Stanford University School of Medicine

C57BL/6J female mice were super-ovulated by intraperitoneal injection of pregnant mare’s serum gonadotropin and human chorionic gonadotropin. These mice were then paired with C57BL/6J males to generate fertilized embryos, and pronucleus (PN) stage embryos were collected. *S. pyogenes* Cas9 protein (IDT) and guide RNAs (target 1 crRNA: CAAACCTAGCCGGATTCCTC, target 2 crRNA: GCACGGTAACCACAAGCTCG, in the form of crRNA:tracrRNA duplex, from IDT) were then electroporated into PN embryos using a NEPA21 electroporation system (Supplementary Fig. 2) with a CUY505P5 electrode. The guide RNAs were designed to delete a 178 bp region at the end of exon 1 and to also introduce an early stop codon in exon 1. This region was deleted because exon 1 is expressed in all 7 known isoforms of *Nav1* mRNAs. Healthy embryos were transferred into the oviducts of pseudo-pregnant recipient females. Genomic DNA from the pups were screened by PCR amplification using the strategy shown in Supplementary Fig. 2. Mice with genomic DNA that generated diagnostic amplicons were subsequently sequenced to characterize the deleted region. To reduce the possibility of off target editing, both selected guide RNAs had very high specificities for the target sites. The target 1 crRNA has an MIT specificity score of 92 and a cutting frequency determination (CFD) specificity score of 96, and target 2 crRNA has an MIT specificity score of 90 and a CFD specificity score of 95^55,56^. Both guide RNA evaluation programs generate scores that range from 0 to 100, with 100 being the most specific. It has been reported that the total number of mismatched base-pairs is a key determinant of Cas9 cleavage efficiency. Two mismatches, particularly those occurring in a PAM-proximal region, considerably reduce Cas9 activity, irrespective of whether they are concatenated or interspaced, and this effect is further magnified for three and four mismatches. Three or more interspaced mismatches eliminates detectable Cas9 cleavage at the vast majority of loci^57^. Of importance, no off-target sites for either crRNA had 0, 1, or 2 base mismatches. The target 1 crRNA has 51 off-target sites with 4 base mismatches and the target 2 crRNA has 41 sites with 4 mismatches; and none are in an exon on chromosome 1. There was one off-target site with 3 base mismatches for both crRNAs; but both sites are in intergenic sequences that were not present on chromosome 1 (where the *Nav1* is located), and at least one mismatch is in the PAM-proximal region. To further minimize the chance of an off-target effect of CRISPR engineering, a *Nav1* KO mouse was backcrossed to C57BL/6J mice for two generations. The resulting *Nav1* het KO mice were intercrossed to generate homozygous *Nav1* KO mice. Since *Nav1* KO females are poor mothers, homozygous *Nav1* KO mice were generated by breeding homozygous *Nav1* KO males with heterozygous *Nav1* females for colony maintenance. To generate mice used in the CSA and FSA experiments, heterozygous *Nav1* mice were intercrossed to generate WT, heterozygous, or homozygous mutant mice. Single molecule fluorescence in situ hybridization (smFISH) was performed according to^58^. In brief, frozen brain tissue sections (20 μm) were pre-treated with 0.01% pepsin in 0.1 M HCl for 2 min at room temperature followed by washing in 0.05% Tween 20 in 1 x diethyl pyrocarbonate-treated (DEPC)-PBS. mRNA was reverse transcribed to cDNA in a room temperature buffer containing 0.5 mM dNTP, 0.2 μg/μl BSA, 1 μM cDNA primer, 1 U/μl RNaseIn (Clonetech, 2313B) and 20 U/μl RT (Maxima, Thermo Scientific™, EP0752) for 3 h at 50 °C in a securely sealed chamber. After three brief washes in 0.05% Tween 20 in 1 x DEPC-PBS, the sections were post-fixed in 4% paraformaldehyde for 30 min at room temperature and washed three times in phosphate-buffered saline with Tween 20 (PBST). Hybridization and ligation with T4 DNA Ligase were performed in T4 ligase buffer with 0.2 μg/μl BSA, 100 nM padlock probe and 0.1 U/μl T4 DNA Ligase (New England Biolabs) for 30–45 min in 37 °C. This was followed by washing with 2× SSC with 0.05% Tween-20 at 37 °C for 5 min, and then rinsing in PBST. Rolling circle amplification (RCA) was performed with 1 U/μl Φ29 DNA polymerase (New England Biolabs) using the reaction buffer supplied by the manufacturer with 250 μM dNTPs, 0.2 μg/μl BSA and 10% glycerol. The incubation was carried out for 60–150 min at 30 °C, which was followed by a washing in TBST. Stranded RCA products (RCP) were hybridized in 250 nM of Cy5 and FAM fluorescence-labeled oligonucleotide probes in a solution of 2× SSC, 20% formamide for 30 min at 37 °C. Slides were then washed in TBST. Dried slides were mounted with VECTASHIELD® PLUS Antifade Mounting Medium (vectorlabs, H-1900-10). Images were acquired using an SP8 Confocal microscope (Leica). For quantification, the number of RCPs and cell nuclei in the images were counted digitally using Fiji software (version 1.53C)^59^. All oligos listed **in** Supplementary Table 5 were synthesized by Integrated DNA Technologies, Inc (Coralville, Iowa).

For proteomic mapping, proteins in brain tissue obtained from C57BL/6J and Nav1 KO (N93) mice were extracted and separated by SDS-PAGE. Protein bands corresponding with the molecular weight of Nav1 were excised, and trypsin digested. The digested peptide mixtures were z-tip purified and run on an Orbitrap Fusion™ Lumos™ Tribrid™ Mass Spectrometer (ThermoFisher, San Jose), which was equipped with an Acquity UPLC M-class system (Waters, MA). The peptide data were searched against the mouse proteome database. Two different Nav1 peptides were found in the C57BL/6J brain sample (MW 202.423; calculated pI 8.06, Score Sequest HT 2.00323, *n* = 2 peptides; and MW 252.876, calculated pI 8.76, Score Sequest 2.131383, *n* = 1 peptide). In contrast Nav1 peptides were completely absent in brain tissue obtained from the *Nav1* KO. Thus, proteomic mapping confirmed that Nav1 protein was absent from brain tissue obtained from homozygous *Nav1* KO mice, while Nav1 peptides were detected in C57BL/6J brain tissue.

### Food self-administration (FSA) assays

The FSA procedure utilized the same testing conditions as CSA, except that 20 µl of Chocolate Boost (Nestle) was delivered as the reinforcer. Additionally, the number of reinforcers per session was not limited to avoid any ceiling effects; the mice had no prior surgical procedures and were not tethered to an infusion line. All sessions were terminated after 2 h of testing. To facilitate acquisition of FSA, home-cage food was removed ~16 h before the 1st FSA session. Following the 1st session, the mice were fed 2.5 g of food in the home-cage, in order to maintain food restriction through the 2nd session. Following the 2nd session, mice were returned to ad lib feeding for the remaining of the testing period. The following numbers of mice of the indicated sex were tested in the FSA assay: *Nav1* KO – 6 F, 5 M; Het – 6 F, 5 M; and Wild type – 6 F, 5 M. The average age of the *Nav1* KO, Het and wild-type mice tested in the FSA assay was 15.8, 15.6 and 14.4 weeks (Supplementary Table 1). Following the 1st FSA experiment, we noted that homozygous *Nav1* KO mice lost more body weight, relative to HET and wildtype mice, in response to food deprivation. Therefore, we tested a second cohort of mice under no food restriction. This cohort was instead subjected to magazine training prior to FSA sessions. This training involved placing mice in the operant chambers and activating a program that turned on the white noise and illuminated the nose-poke apertures and house light. After 10 s, 20 µl of Boost was delivered to the magazine, the aperture lights turned off and the house light flashed (1 s on, 1 s off). These conditions continued until the mouse entered the magazine, as detected by infrared beam break. Entry to the magazine stopped the flashing house light and illuminated the apertures. 30 s following the magazine entry, 20 µl of Boost was again delivered to the magazine, the aperture lights turned off and the house light flashed until another magazine entry. The sessions were limited to 50 Boost rewards or 2 h. Mice were required to earn at least 30 rewards before moving on to FSA testing.

After 10 days of FR1 testing, mice in the CSA and FSA studies were tested under a progressive ratio schedule of reinforcement in one session. The first reinforcer required one press of the active lever, and the response requirement was doubled every reinforcer thereafter. The session ended when the mouse failed to earn a reinforcer in 30 min, or 2 h total elapsed. The last ratio achieved for each mouse and was utilized as an indicator of performance.

### Analysis of the CSA and FSA data for assessing the effect of the Nav1 KO

C57BL/6J (Wt), heterozygous *Nav1* KO (Het) and homozygous *Nav1* KO mice (KO), which were all naive to any prior experimentation, were tested for CSA using a between-subjects dose-response test with cocaine doses of 0.1, 0.5, and 1.0 mg/kg. To determine if sex impacted the *Nav1* KO effect on CSA, male and female mice were tested. For all CSA and FSA studies involving *Nav1* KO mice, het-het breeding pairs were used to generate offspring of all three genotypes, including the wild-type mice that were utilized as controls. The following numbers of mice of the indicated sex were tested at each of the following indicated doses: *Nav1* KO – 0.1 Dose: 7 F, 7 M; 0.5 Dose: 8 F, 8 M; 1.0 Dose: 6 F, 8 M; Het – 0.1 Dose: 7 F, 8 M; 0.5 Dose: 7 F, 8 M; 1.0 Dose: 6 F, 7 M; and Wildtype – 0.1 Dose: 7 F, 8 M; 0.5 Dose: 7 F, 9 M; 1.0 Dose: 8 F, 6 M. The average age of the *Nav1* KO, Het, and wild-type mice tested in the CSA assay was 16.2, 16.2, and 16.3 weeks. For the FR1 CSA data, the results for the effect of a variable (i.e., mouse genotype, sex, or the effect of an individual session) on the number of cocaine infusions are reported as an F-statistic [F(variation among sample means / variation within sample)] and as a *p*-value. Variables that had a significant effect were further evaluated by performing pairwise comparisons of that variable (i.e., genotype or sex) with the CSA results obtained at each session, and the results are reported as a *p*-value for the effect of that variable on the number of cocaine infusions.

### Acute locomotor effects of cocaine

Mice, which were naïve to any prior experimentation, were tested in an acute locomotor dose-response procedure (*Nav1* KO – 6F, 6M; Het – 6F, 5M; and Wild type – 6F, 6M). The average age of the *Nav1* KO, Het and wild-type mice tested in the cocaine locomotor assay was 14.7, 14.2, and 14.3 weeks. Testing occurred in Med Associates open field boxes (43.2 × 43.2 × 30.5 cm, Med-Associates MED-OFAS-RSU; St Albans VT), housed individually in sound-attenuating chambers. All testing sessions were preceded by a 30-min acclimation period in the open field box with no prior injection. Following this 30-min session, mice were briefly removed and received an intraperitoneal (ip) injection of either saline (sessions 1 and 2) or cocaine (sessions 3, 4, and 5) at a dose volume of 10 mL/kg body-weight, and returned to the open field box for 1 h. Cocaine was administered at 3 doses (5, 10, and 20 mg/kg body weight) in a within-subjects dose-response design. All 6 possible dose orders were utilized and balanced across genotype groups. Sessions 1, 2, and 3 occurred over 3 consecutive days. Sessions 4 occurred 2 days following session 3 and session 5 occurred 3 days following session 4, in order to limit any potential effects of prior cocaine exposures.

Locomotor behavior was assessed by distance traveled, as determined by infrared beam breaks. Distance traveled under no injection, saline injection, and cocaine injection was assessed. The acute locomotor effect of cocaine was calculated by subtracting the average of the distance traveled after both saline injection sessions from the distance traveled after cocaine injection. The data were binned into two, 30-minute bins and assessed within bin in addition to the full 1-h session.

### MRI analyses

The brains of age-matched adult male C57BL/6J and *Nav1* KO mice (*n* = 4/group, age 3–4 months) were examined by in vivo MRI using a high-field 7 T MRI scanner (Bruker, Billerica, MA) at the Stanford Center for Innovation in In vivo Imaging (SCi^3^) facility. All mice were anesthetized under 1.0–1.5% isoflurane that was administered by nose cone throughout the session. Their body temperatures were supported with warm air, while their respiratory rates were continuously monitored. Anatomical images were acquired using T2-weighted turbo rapid acquisition with relaxation enhancement (T2 TurboRARE) with the following parameters: repetition time (TR) = 2500 ms, echo time (TE) = 40 ms, flip angle = 90 degrees, slice thickness = 0.5 mm. Slices were obtained in the axial view (coronal in the mouse) with the first slice starting at the rostral-most extension of the prefrontal/motor cortex, while the olfactory bulb was excluded. The DICOM files obtained were processed using Osirix software (Pixmeo SARL, Bernex, Switzerland). The cortical thickness, hippocampal volume, and brain volume were manually labeled by an experimenter that was blinded to the genotype of mice. Measurements were obtained from a continuous series of slices (for cortical thickness: from +0.63 to −0.67 mm; for hippocampal volume: −0.77 to −3.37 mm; for brain volume: +3.33 to −4.87 mm; locations relative to Bregma) that were aligned across mice groups. The normalized hippocampal volume was calculated by dividing the absolute hippocampal volume by the total brain volume. The MRI data were analyzed using Prism 9.1.0 (GraphPad Software, Inc. La Jolla, CA) with unpaired student *t*-test.

### Whole-cell patch-clamp recording

Brain slices were prepared from anesthetized male mice (3–4 months of age) using previously described techniques^60^. In brief, coronal slices (~300 μm) were prepared from excised brains that were sectioned with a vibratome in cold (4 °C) buffer (ACSF) used for tissue slicing that contains: 126 mM NaCl, 2.5 mM KCl, 1.25 mM NaH_2_PO_4_, 1 mM CaCl_2_, 2 mM MgSO_4_, 26 mM NaHCO_3_, and 10 mM glucose; pH 7.4, when saturated with 95% O_2_/5% CO_2_. Slices were then transferred to an incubation chamber filled with standard ACSF buffer containing: 126 mM NaCl, 2.5 mM KCl, 1.25 mM NaH_2_PO_4_, 2 mM CaCl_2_, 1 mM MgSO_4_, 26 mM NaHCO_3_, and 10 mM glucose. The slices were incubated at 33 ± 1 °C for 1 h, and then at room temperature before use. After incubation, slices were transferred to a recording chamber where they were minimally submerged (32 ± 1 °C) and perfused at the rate of 2.5–3 mL/min with standard ACSF buffer. Patch electrodes pulled from borosilicate glass tubing (1.5 mm OD) and had impedances of 4–6 MΩ when filled with Cs-gluconate based intracellular solution containing: 120 mM Cs-gluconate, 10 mM KCl, 11 mM EGTA, 1 mM CaCl_2_, 2 mM MgCl_2_, 10 mM HEPES, 2 mM Na_2_ATP, 0.5 mM NaGTP. The osmolarity of the pipette solution was adjusted to 285–295 mOsm and the pH to 7.35–7.4 with CsOH and E_Cl_^−^ was −70 mV calculated from the Nernst equation. Whole-cell voltage clamp recordings of miniature (m) IPSCs were obtained from the granule cells in the dentate gyrus of right hippocampus at a holding potential (V_h_) of +20 mV in the presence of 1 μM tetrodotoxin (TTX, Ascent Scientific) without application of glutamate receptor blocking agents^60^. Miniature (m) EPSCs were recorded from the granule cells at *V*_h_ = −70 mV, the estimated E_Cl_^−^ with the Cs-gluconate internal solution^60^. All recordings were made with a Multiclamp 700 A amplifier, sampled at 10 kHz, filtered at 4 kHz with a Digidata 1320 A digitizer, and analyzed using Clampfit 9.0 (Molecular Devices, Sunnyvalle, CA), Mini Analysis (Synaptosoft, Decatur, GA), and Prism (GraphPad software). Only recordings with a stable access resistance <20 MΩ that varied <15% during the recording were accepted for analysis. One or two neurons were recorded per slice, and no more than three slices were used per mouse.

### Tissue collection and sectioning

After isoflurane anesthesia, transcardial perfusion (Harvard Apparatus p-70, Holliston, MA) was performed with a 0.15 M NaCl solution was followed by fixation in 4% paraformaldehyde (Aldrich Chemistry, Darmstadt, Germany). The brains were then extracted and post-fixed in 4% paraformaldehyde for 4 h. After fixation, the brains were transferred into a 30% sucrose solution, and then stored at 4 °C until sectioning. The brain tissue slices used for immunohistochemistry were sectioned at 30 µm, and then stored in a cryo-protective solution (PBS, 20 g PVP-40, 600 ml ethylene glycol, 600 g sucrose) at −20 °C.

### Immunohistochemistry

The immunohistochemical analyses were performed as described in ref. ^61^. In brief, brain sections were rinsed 5 times in PBS (Sigma-Aldrich P5368-10pak) for 5 min, and then blocked in 10% normal donkey serum and 0.3% Triton X-100 in PBS to minimize nonspecific binding. The sections were then incubated with the primary antibodies (Supplementary Table 4) in the blocking solution for 48 h at 4 °C. The sections were then rinsed in PBS and incubated with a corresponding secondary antibody for 4 h. After the last rinse, the sections were mounted, air dried overnight; and were then sealed with a cover slip in Dako Fluorescence Mounting Medium (S3023, Dako North America, Inc., Carpinteria, CA) and stored at −20 °C.

### Image capture

Images were captured and analyzed using a Leica SP8 white light pulsed laser confocal microscope and Leica LAS X Premium software at the Cell Sciences Imaging Facility at Stanford. Channels were selected and the exposure times were adjusted to optimize the images. Imaris software (Bitplane Inc., Concord, MA) was used for image capture^62^.

### Analysis of learning, memory, and exploratory behaviors

Pre-experiment habituation was performed on all mice used in the behavioral tests described below; this involved daily handling and habituation that was initiated a week before the behavioral testing began. Mice were picked up by hand, stroked and touched for approximately 2 min per session. A quick assessment was made during the last handling session; if a mouse exhibited high levels of anxiety-like behavior (incontinence, hyperactivity, etc.), 1–2 additional handling sessions were conducted before experimental testing.

### Open field test

The open field test was conducted as described^63^ using a 40 × 40 × 40 cm (l × w × h) square arena with white plastic boards. Each mouse was habituated within the procedure room for 30 min and was then placed in the center of the open field arena. The total distance traveled and duration of time in the center (~25% of total area) or edge over a period of 15 min were recorded and analyzed using Viewer III software (BIOBSERVE, Bonn, Germany). The total duration of the open field test was 15 min.

### Novel object recognition test

The novel object recognition test was conducted as described^64^ in the apparatus used for the open field tests. A test mouse was given free access to the entire chamber for a 5 min habituation period. When the training session started, two identical objects (Lego blocks or a flask filled with bedding) were placed in diagonally opposite corners of the arena (6 cm from the wall), and the test mouse was allowed to freely explore for 10 min. After 24 h, the test mouse was returned to the center of the arena and habituated for 5 min. When the testing session started, one familiar and one novel object were presented at the same positions in the arena. The test mouse was then given 10 min to explore the objects, while exploratory behaviors (sniffing, rearing against the objects, and head within 2 cm of the object) were recorded. Videos were processed using Viewer III tracking software by experienced personnel. The first 5 min of the training and testing sessions were used for analysis. The discrimination index (DI) was calculated as:

1 $$({{{{{\rm{Exploration}}}}}}\; {{{{{\rm{time}}}}}}\; {{{{{\rm{with}}}}}}\; {{{{{\rm{novel}}}}}}\; {{{{{\rm{object}}}}}}-{{{{{\rm{Exploration}}}}}}\; {{{{{\rm{time}}}}}}\; {{{{{\rm{with}}}}}}\; {{{{{\rm{familiar}}}}}}\; {{{{{\rm{object}}}}}})/ \\ \left(\right.{{{{{\rm{Exploration}}}}}}\; {{{{{\rm{time}}}}}}\; {{{{{\rm{with}}}}}}\; {{{{{\rm{novel}}}}}}\; {{{{{\rm{object}}}}}}+{{{{{\rm{Exploration}}}}}}\; {{{{{\rm{time}}}}}}\; {{{{{\rm{with}}}}}}\; {{{{{\rm{familiar}}}}}}\; {{{{{\rm{object}}}}}}$$

### Elevated plus maze test

The elevated plus maze test was conducted as described^63^ in an arena with two open arms and two closed arms that are raised 50 cm above the floor. All arms were 30 × 5 cm (l × w) with white walls (15 cm height) and floors. The test started 2 h after the end of the dark cycle. Thirty minutes after acclimation in the behavioral room, the test mouse was placed in the center of the maze and allowed to freely explore the arena. The total test duration was 5 min. The duration of time spent in exploratory behaviors, the number of entries and the distance traveled in the open and closed arms (excluding center) were analyzed using Viewer III tracking software.

### Rotarod test

Motor coordination was evaluated using methods that are described in the Standard Operating Procedures of the Jackson Laboratory Mouse Neurobehavioral Phenotyping Facility using a five-station rotarod treadmill (ENV-575M, Med Associates, St. Albans, VT). Mice were first acclimated to the behavioral room for 1 hr before testing. The testing session consisted of three trials; in each trial, the speed was increased from 4 to 40 rpm; and each trial was separated by an interval of 1 min. A trial was terminated when a mouse fell off, clung to the rod and completed full passive rotation, or after 300 s. The duration and end speed on rotarod were recorded and averaged from the last two trials for each mouse.

### Barnes maze test

Barnes maze tests were conducted as described in ref. ^65^. The experimental protocol consisted of a habituation session (day 1); 12 training sessions with 3 trials per day and a 15 min intertrial interval on days 2–5; and the testing session on day 6. During the training sessions, the mice were released into the middle of the maze, and they learned to enter the open escape hole to avoid exposure to a strong light. Three visual cues were placed at distinct positions outside of the maze to facilitate learning. The maze was cleaned with 70% ethanol thoroughly between trials to eliminate olfactory cues. Twenty-four hours after the training sessions, mice were tested in the arena for 90 s with all holes closed. The results evaluated include primary errors (errors made before reaching the escape hole), latency (the time elapsed before reaching the escape hole), track length (the total length traveled), and target hole preference (percentage of time spent adjacent to the escape hole). Their performance was recorded and analyzed using Viewer III tracking software.

### Statistics and reproducibility

Prism 9.1.0 was used for analysis of the MRI data and Prism 8.4.1 for Windows (GraphPad Software, Inc. La Jolla, CA) was used for analyzing the behavioral data. Other data were analyzed using Statistica 8.0 (TIBCO Software, Inc. Palo Alto, CA) or SPSS (IBM Corp. Released 2020. IBM SPSS Statistics for Windows, Version 27.0. Armonk, NY: IBM Corp). The CSA, FSA, and acute cocaine locomotor behavior results were assessed by ANOVA. Interactions and main effects were decomposed by simple main effects and pairwise comparisons with Sidak correction for multiple comparisons. Since CSA data tends to depart from normal distributions, all CSA data was log_10_ transformed prior to analysis. For the acute locomotor response, Barnes maze test, and novel object recognition test, the statistical significance was determined using a two-factor ANOVA with repeated measures. Stimulus (familiar vs novel) or genotype (control vs. KO) were the two factors assessed. Post-hoc analysis was conducted using a Bonferroni post-test. Exploratory behaviors were evaluated using a one-way ANOVA with Tukey’s post-test. In the figures, the significance levels are indicated by: *p < 0.05, **p < 0.01, ***p < 0.001, ****p < 0.0001.

### snRNA-Seq analysis

The protocol used for nuclear isolation was modified from that developed by 10X Genomics. Brain tissue was obtained from age-matched adult male *Nav1* KO (*n* = 4) and C57BL/6J (*n* = 5) mice. The PFC was quickly dissected from the freshly extracted brain tissue and it was place in chilled Hibernate AB Complete (HEB) medium (BrainBits LLC, Springfield, IL) at 4 °C. Freshly dissected PFC from each group were pooled in separate 50 ml conical tubes with a minimum amount of chilled Hibernate AB Complete (HEB) medium (BrainBits LLC, Springfield, IL). Then, 5 ml of chilled lysis buffer (2 mM Tris-HCl, 2 mM NaCl, 0.6 mM MgCl_2_ and 0.02% Nonidet^TM^ P40 Substitute in nuclease-free water) was added to the tubes, which were then incubated at 4 °C for 10 min. The amount of lysis buffer and the incubation time were optimized determining the amount of buffer and incubation time that produced high-quality nuclei. After adding 5 more ml of HEB medium to the tubes, the tissues were triturated with a fire-polished silanized Pasteur pipette for 10–15 passes and then strained with 30 μm MACS strainer (Miltenyi Biotec Inc, Auburn CA). Nuclei were pelleted by centrifugation at 500 × *g* for 5 min at 4 °C and were then resuspended in a chilled PBS with 1% BSA (Invitrogen AM2618, Thermo Fisher Scientific, Pittsburgh PA) wash buffer with 0.2 U/ul RNase inhibitor (#3335402001, Millipore Sigma, Darmstadt Germany). The nuclei were pelleted and resuspended twice, strained using a 30 μm MACS strainer, and then centrifuged and resuspended in a chilled wash buffer. Mouse anti-NeuN antibody (MAB377, Millipore Sigma, Darmstadt Germany) was added at a 1:500 dilution, and the samples were incubated for 40 min at 4 °C. Samples were then centrifuged at 400 × *g* for 5 min at 4 °C and the pelleted nuclei were resuspended with chilled wash buffer. Alexa 647 chicken anti-mouse antibody (A21463, Life technologies, Pittsburgh PA) was added to the preparations at a 1:500 dilution and the samples were incubated for 40 min at 4 °C. After incubation, nuclei were pelleted and resuspended, and Hoechst 33342 (Invitrogen H3570, Thermo Fisher Scientific, Pittsburgh PA) was added to a final concentration of 0.25 μg/ml.

Single nuclei sorting was conducted using a 6-laser BD Influx sorter (BD Biosciences, San Jose CA) with a 100-um nozzle in the Stanford Shared FACS Facility. Various control samples (unstained, Hochest33342^+^ or NeuN^+^) were examined to optimize the gating strategy. Nuclei were gated based on size, scatter properties and staining for Hoechst and NeuN. To ensure that we were able to analyze different types of cells, ~60% of the sorted nuclei were collected as NeuN^+^ and 40% were NeuN^-^. Single nuclei were sorted into collecting tubes and then visually inspected under a microscope for quality control; they were then pelleted and resuspended to produce a solution with 600 nuclei/ul in the final volume. Single nuclei were then captured in droplets with barcodes using the 10x Genomics Chromium system and cDNA libraries were produced using Chromium Next GEM Single Cell 3’ Reagent Kits v3.1 (10x Genomics, Pleasanton, CA) according to the manufacturer’s instructions. The *Nav1* KO and C57BL/6J samples were sequenced on the NovaSeq platform (Illumina, San Diego CA).

FASTQ files with the snRNA-Seq data were processed using Cell Ranger software (v6.0.0) and the *cellranger count* pipeline to generate a filtered feature-barcode matrix (the gene expression matrix). The reads within the C57BL/6J and *Nav1* KO FASTQ files were aligned using the Cell Ranger built-in mouse reference (mm10). Two gene expression matrices were produced: 23445 features × 15402 cells for C57BL/6; and 23737 features × 14623 cells for *Nav1* KO. The C57BL/6 J and *Nav1* KO gene expression matrices were then analyzed using the R/Seurat (v4.0.1) package. Low-quality cells with unique gene counts <200 and >10% mitochondrial counts were filtered out. The high-quality C57BL/6J (20955 features × 14,630 cells) and *Nav1* KO (21,310 features × 14,056 cells) matrices were merged into one Seurat object (21,892 features × 28,686 cells) that was used for subsequent analyses (data normalization, variable feature identification, dimensional reduction, etc.).

The gene counts for each cell within the global matrix was normalized by dividing it by the total counts in that nucleus; and this number was multiplied by 10,000 and were then natural log transformed to become the normalized values. We also identified 2000 variable features, which exhibited high cell-to-cell variability in the matrix. The matrix was then further scaled for linear dimensional reduction purposes. For the scaled gene expression matrix, the mean expression of a gene across different cells is set to 0 and the variance is set to 1. Principal component analysis (PCA) was performed on the scaled data, and the first 10 PCs were chosen to represent the dimensionality of the global matrix. The default K-nearest neighbor (KNN) graph-based method and Louvain algorithm for single-cell clustering was used; and the resolution parameter was set to 0.3. In total, 14 clusters (cluster 0 to 13) were classified; and of these, cluster 0 contained the most cells while cluster 13 contained the fewest. To visualize the cells in low-dimensional space (to aid interpretation), the non-linear dimensional reduction program (UMAP)^66^ was performed using the first 10 PCs.

The differentially expressed genes (DEG) for each cluster were detected using a minimum percentage of 0.25 in either of the two clusters and an average log_2_ fold-change (FC) ≥ 0.25. Cell type-specific canonical markers were used to determine the cell type identity of the 14 clusters. Non-neuron cells were assigned as follows: Astrocyte (*Gja1, Aqp4*), Microglia (*C1qa*), and Oligodendrocyte (*Apsa, Mbp*). Of note, no endothelial cell markers (*Flt1, Cldn5, Nostrin*) were highly expressed in any of 14 clusters. The type of neuronal cells was determined by whether they expressed mRNA markers for excitatory (*Slc17a7, Tshz2, Thsd7a*) or inhibitory (*Gad1, Gad2, Slc32a1, Meis2*) neuronal markers. To verify the cell type assignments, our C57BL/6J gene expression matrix was compared with that of a published reference (GSE124952)^32^ data set for PFC cells obtained from saline control C57BL/6J mice, which generated an expression matrix with 20718 features × 11886 cells. The canonical correlation analysis (CCA) between this reference and our C57BL/6J dataset was used to remove batch effects before matrix integration. We then projected the reference single cells with their cell type labels onto the UMAP plot for comparison with our C57BL/6J dataset.

### Reporting summary

Further information on research design is available in the Nature Portfolio Reporting Summary linked to this article.

### Supplementary information

Supplementary Information

Description of Supplementary Files

Supplementary Data 1

Reporting Summary

## Data Availability

All raw single cell RNA-seq data and the processed data were deposited in the Gene Expression Omnibus (GEO): GSE216957. SNP allele data is also available at the Mouse Phenome Database (GenomeMUSter https://mpd.jax.org/genotypes). The raw data for Figs. 1a, d, and 2, 3 are available in Supplementary Data 1.
