# Supplementary material for: Neuron Navigator 1 (Nav1) regulates the response to cocaine in mice
Source: Commun Biol. 2023 Oct 18;6:1053. doi: 10.1038/s42003-023-05430-9 (PMC10584906; doi:10.1038/s42003-023-05430-9)
Supplement: Supplementary file 1 — Supplementary Information [file 42003_2023_5430_MOESM1_ESM.pdf]

Supplementary Information

Neuron Navigator 1 (Nav1) Regulates the Response to Cocaine in Mice

Genotype	Average Age (wks)	Max Age (wks)	Min Age (wks)	n
**CSA**				
Het	16.2	33.1	10.1	41
KO	16.2	32.4	9.3	44
Wt	16.3	32.4	9.3	46
**FSA**				
Het	15.6	17.6	14.7	11
KO	15.8	17.9	14.7	11
Wt	14.4	17.6	10.3	11
**Cocaine Locomotor**				
Het	14.2	25.1	9.3	12
KO	14.7	25.1	8.7	12
Wt	14.3	21.4	9.4	11

Supplementary Table 1. The average ages of the wildtype, het and *Nav1* KO mice used in the CSA, FSA and cocaine locomotor tests.

Gene	HBCGM p-value	PS p-value
Tnni1	1.3×10^{-06}	0.16
Etnk2	1.8×10^{-06}	0.0032
Nav1	3.4×10^{-06}	**0.26**
Ipo9	4.2×10^{-06}	0.040
Sh3pxd2a	5.1×10^{-06}	0.11
Cntn2	8.5×10^{-06}	0.49
Chi3l1	1.2×10^{-05}	0.13

Supplementary Table 2. The results of association tests for population structure (PS) that were performed on haplotype blocks within the indicated genes are shown. Because of ancestral relationships among the inbred strains, a GWAS using inbred strains can generate false positive genetic associations because commonly inherited genomic regions could randomly correlate with phenotypic trait values. Therefore, PS association tests were performed for the indicated candidate genes to determine if the strain groupings within the haplotype blocks could result from commonly inherited genomic segments (based upon the overall similarity of shared alleles in their genomes) using the method described in [1]. A PS p-value >0.05 indicates that the alleles within the haplotype block for the indicated gene do not reflect population structure. The p-value for the genetic association with the CSA data, which was calculated by the HBCGM program, is also shown for each haplotype block.

Supplementary Table 3. Synonymous and nonsynonymous SNPs within the coding sequence of *Nav1***.** The nucleotide position, the affected amino acid, and the C57BL/6 and the alternative (ALT) amino acids are shown for each SNP. The three cSNPs are highlighted in bold.

Chr	Genomic Pos	Amino Acid	C57BL/6	ALT
1	135584853	156	S	S
1	135584727	**198**	**D**	**E**
1	135532666	306	S	S
1	135532552	344	G	G
1	135532354	410	T	T
1	135472430	467	S	S
1	135471052	597	S	S
1	135470854	663	L	L
1	135470761	694	R	R
1	135470033	799	S	S
1	135469724	902	S	S
1	135469698	**911**	**A**	**V**
1	135454498	1319	S	S
1	135454067	**1366**	**P**	**L**
1	135450851	1567	D	D
1	135450848	1568	L	L
1	135450076	1618	S	S
1	135450064	1622	E	E
1	135449968	1654	L	L
1	135449036	1683	F	F
1	135448967	1706	I	I
1	135448937	1716	G	G
1	135441768	1788	L	L

Primary Antibody	Cat #	Vendor	Dilution
guinea pig anti-vGlut1	135304	Synaptic Systems	1/1000
rabbit anti-PSD95	51-6900	Invitrogen	7/1000
guinea pig anti-vGat	131004	Synaptic Systems	1/1000
mouse anti-gephyrin	147021	Synaptic Systems	1/400
rabbit anti-MAP2	AB5622	millipore	1/1000
Mouse anti-tubulin	801201	BioLegend	1/1000

Supplementary Table 4. The primary antibodies used for immunohistochemistry, their source and the dilution used in an experiment are shown.

RT_Nav1_wt _woLNA	Ttgctggaaagcagttcgtc
RT_Nav1_Del_woLNA	Tctgtcggagatccagaacg
Nav1wt	gtcaagcctctcagcaaggcgTCGTCTTAATCACTAGTCGGAAGTACTACTCTCTTACGCTTACAACTAGctcggggaccctatgcggag
Nav1-178	GATTCgcttgtggttaccgtgctACTTTCTATGATTACTGACCTACCTCAATGCACATGTTTGGCTCCTCTTCagcctaggcaaacctagccg
DP-5	/5Cy5/AGTCGGAAGTACTACTCTCT
DP-2	/56-FAM/CCTCAATGCACATGTTTGGCTCC

Supplementary Table 5. The oligonucleotide probes used for smFISH analysis are shown.

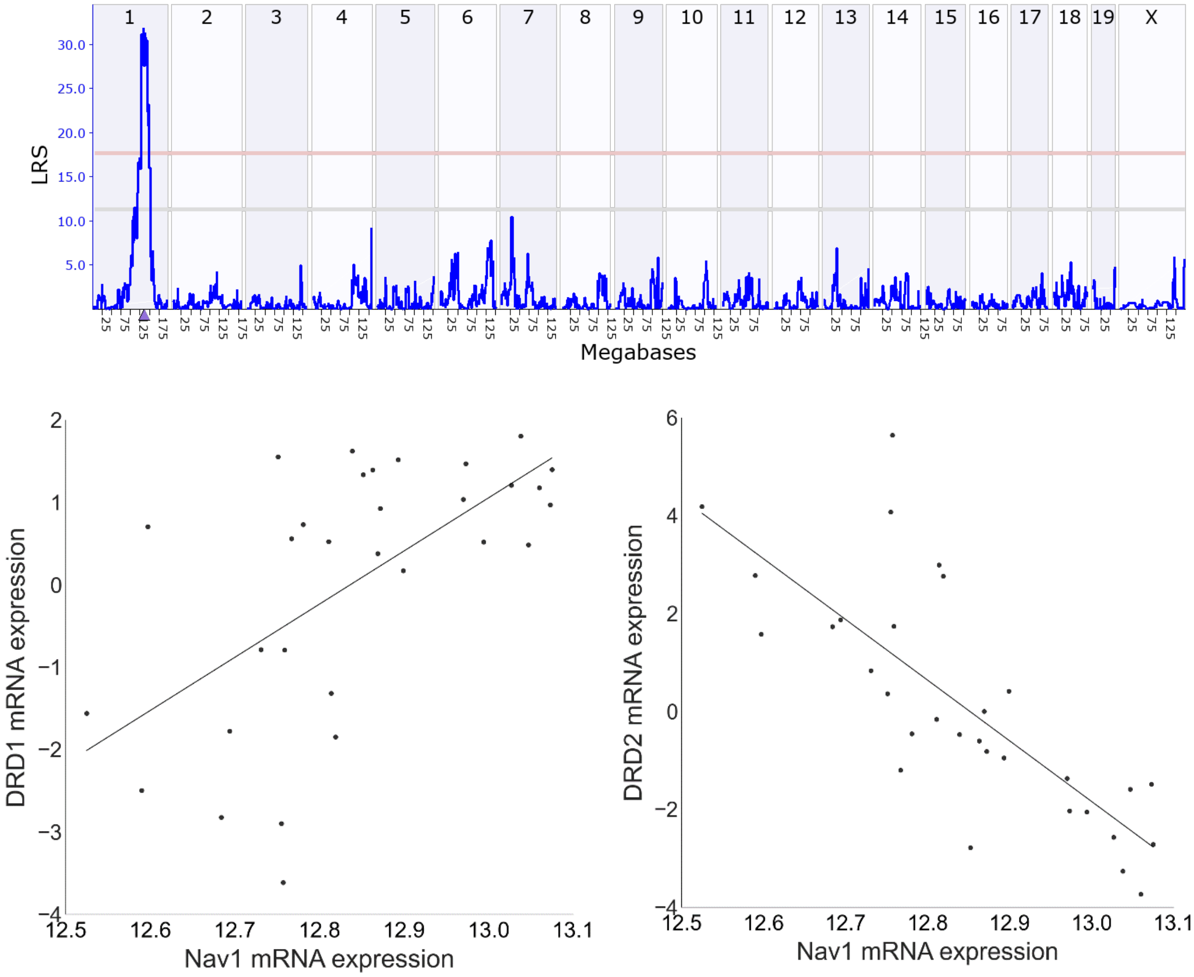

Supplementary Figure 1. Evidence supporting Nav1 as a candidate gene for CSA. (**a**) The level of striatal *Nav1* mRNA expression in adult BXD mice (genenetwork.org; record ID ILM6620129) is controlled by cis-acting alleles within the *Nav1* locus (Chr 1 135.4 MB). The red horizontal line indicates the genome-wide significance threshold for an LRS/LOD score as determined by permutation analysis. (**b,c**) The *Nav1* eQTL was evaluated for genetically correlated phenotypes measured in the BXD database (genenetwork.org). The level of correlation of *Nav1* mRNA expression with the entire database of BXD phenotypes was evaluated. *Nav1* mRNA expression was most highly correlated (inverse) with the level of striatal *dopamine receptor D2* (*Drd2*) mRNA expression (record ID: 15186; r=-.78; p=4.23x10^{-8}). The *Drd2* mRNA correlation is quite specific because striatal *Drd1* mRNA expression is positively associated (record ID: 15186; r=.61; p=1.81x10^{-4}) with the level of *Nav1* mRNA expression.

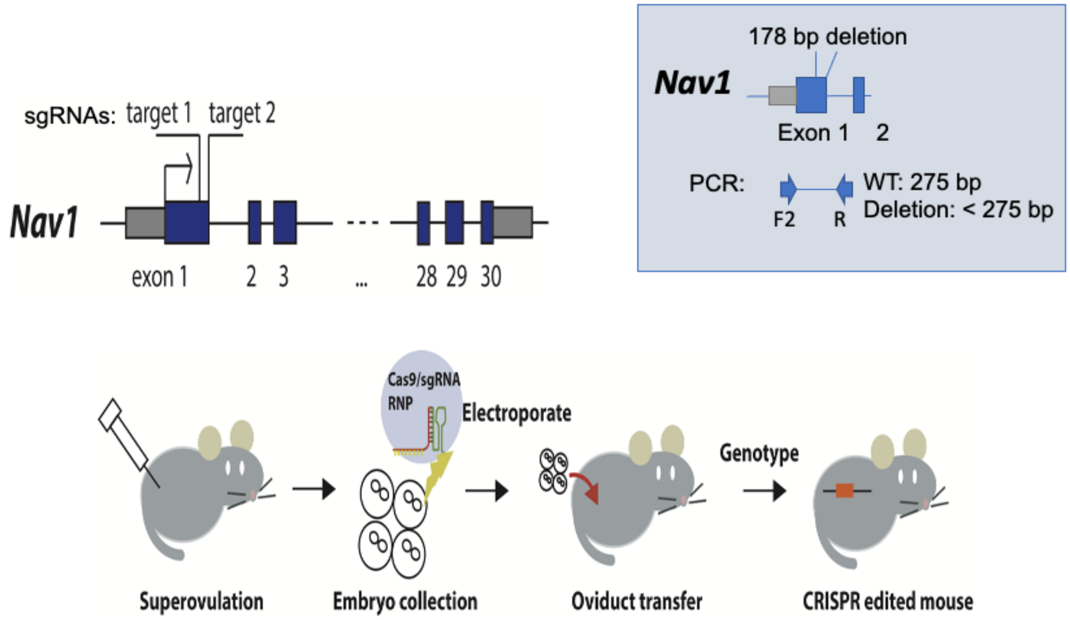

Supplementary Figure 2. CRISPR-mediated genome engineering was used to produce *Nav1* knockout mice**.** C57BL/6 female mice were super-ovulated by intraperitoneal injection of pregnant mare serum gonadotropin and human chorionic gonadotropin. These mice were paired with C57BL/6 males to generate fertile embryos, and pronucleus stage embryos were collected. Then, Cas9, and the two sgRNAs (shown above) were electroporated into embryos. These sgRNAs were designed to delete a 178 bp region at the end of exon 1 in *Nav1*, and it also introduced an early stop codon into exon 1. Healthy embryos were then transferred into the oviducts of pseudo-pregnant recipient females. Genomic DNA from the resulting pups is screened by PCR amplification using the strategy shown in the colored box. While an intact *Nav1* gene will produce a 275 bp amplicon, genomic DNA from an engineered mouse will generate a shorter amplicon that is diagnostic of a deletion. Mice with a heterozygous *Nav1* KO were then bred to produce homozygous *Nav1* KO mice.

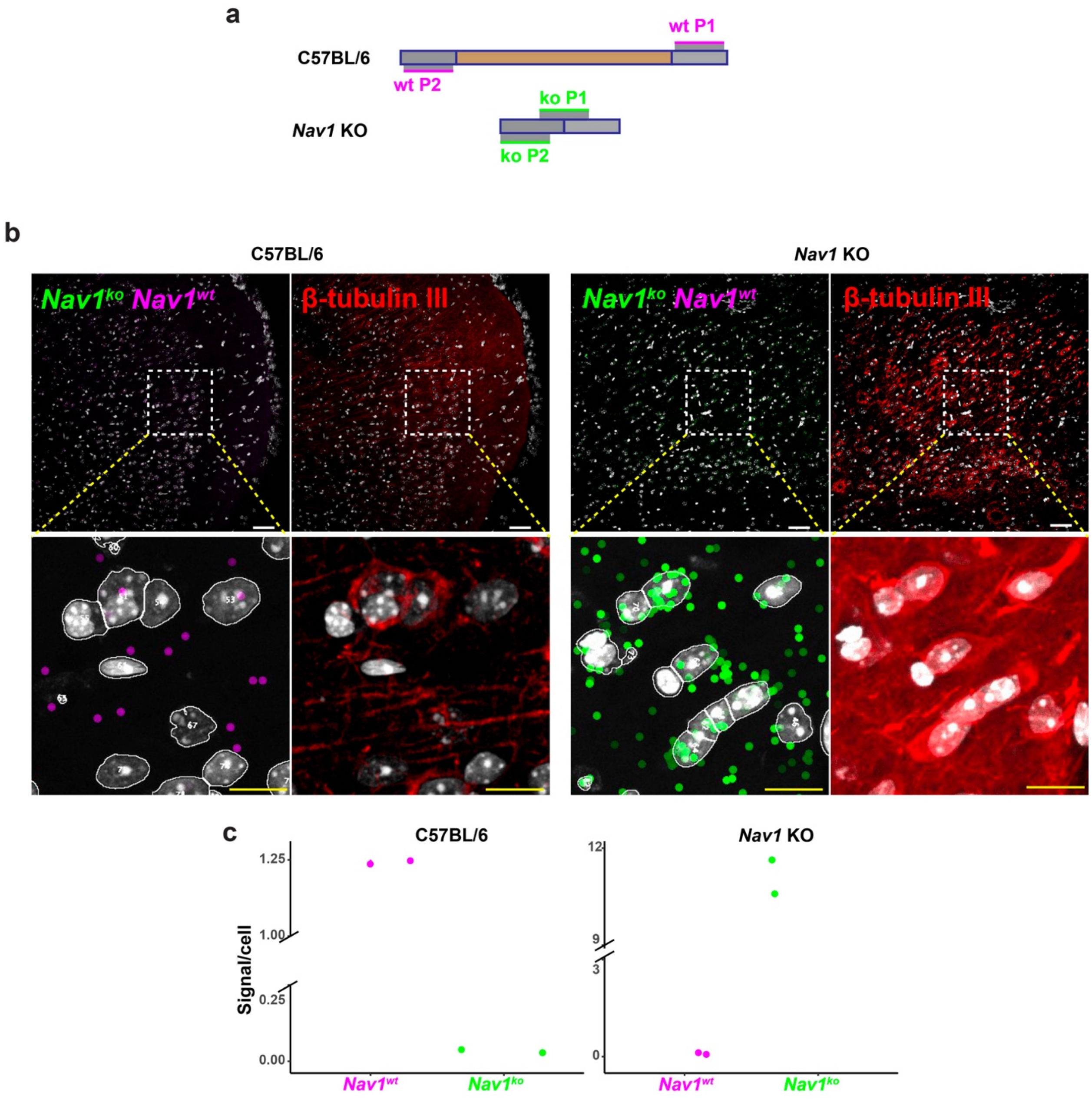

Supplementary Figure 3. *Nav1* mRNA is not expressed in *Nav1* KO mice. **(a)** An illustration of the genomic regions within Exon 1 of *Nav1* recognized by the padlock probes that are specific for wildtype *Nav1* or *Nav1* KO mRNA [2]. The gray boxes represent the 5' and 3' regions of exon 1 and the brown box represents the -178 bp deletion that is introduced into exon 1 to produce the *Nav1* KO. The wildtype *Nav1* probe (Nav1 wt) binds to sites (wtP1, wtP2) present in the wildtype *Nav1* allele; while the *Nav1* KO probe binds to adjacent regions around the 178 bp deletion (koP1, koP2), which are specific for the *Nav1* KO allele. **(b)** sm-FISH images obtained using the *Nav1* and *Nav1* KO probes on coronal sections of prefrontal cortex (PFC) tissue

obtained from C57BL/6 and *Nav1* KO mice. The sections were also stained with anti-β-tubulin (β-Tubulin III) antibodies to identify neurons. The top panels show low power images; and the bottom panels are high power images of the boxed region shown in the top row. The scale bars are 50 μm in the upper images, and 10 um in the lower images. The staining pattern indicates that wild type *Nav1* mRNA is expressed in the PFC of C57BL/6 but not in *Nav1* KO mice, while the *Nav1* KO transcript is exclusively expressed in *Nav1* KO mice. **(c)** The single molecule counts generated for each padlock probe in the C57BL/6 and *Nav1* KO brain sections are shown. Each group has 2 independent measurements.

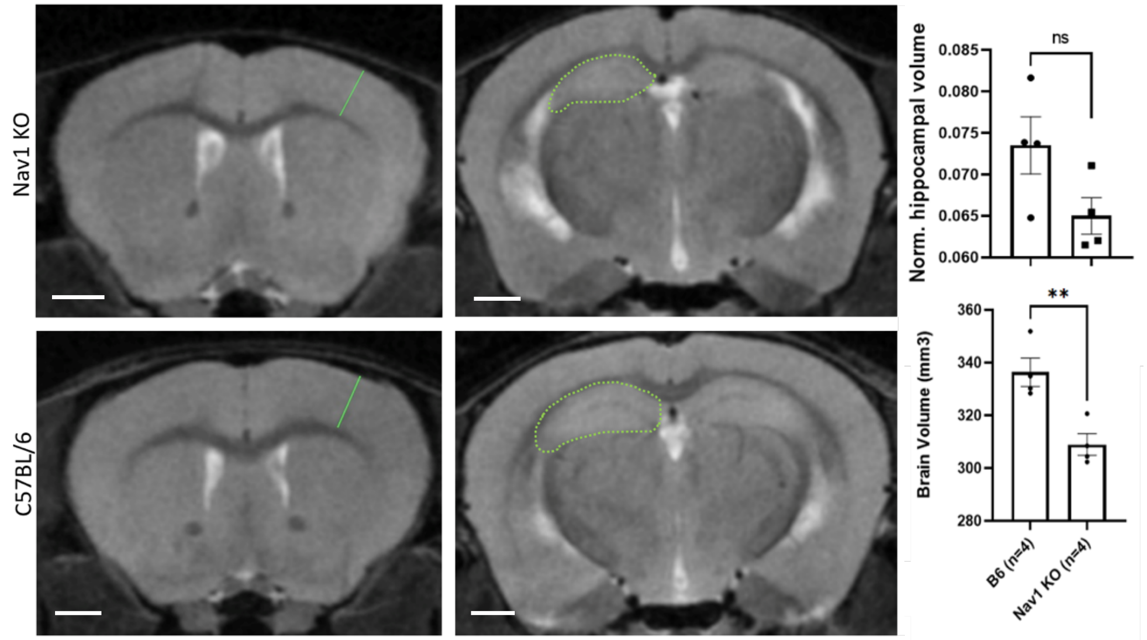

Supplementary Figure 4. MRI scans of coronal sections of *Nav1* KO and age-matched isogenic C57BL/6 mice that were obtained with a Bruker 7-T MRI. The cortical thickness (solid line) and hippocampal volume (dotted line) measurements were generated as indicated in the images. In the adjacent graphs, each bar is the average ± SEM measurements made on *Nav1* KO and C57BL/6 (n=4 per group) mice. *Nav1* KO mice have a slightly smaller overall brain volume than C57BL/6 mice (p=0.007). However, after normalization relative to brain volume, there was not a significant difference (p=0.08) between the hippocampal volumes of C57BL/6 and *Nav1* KO mice. (White scale bar: 1 mm).

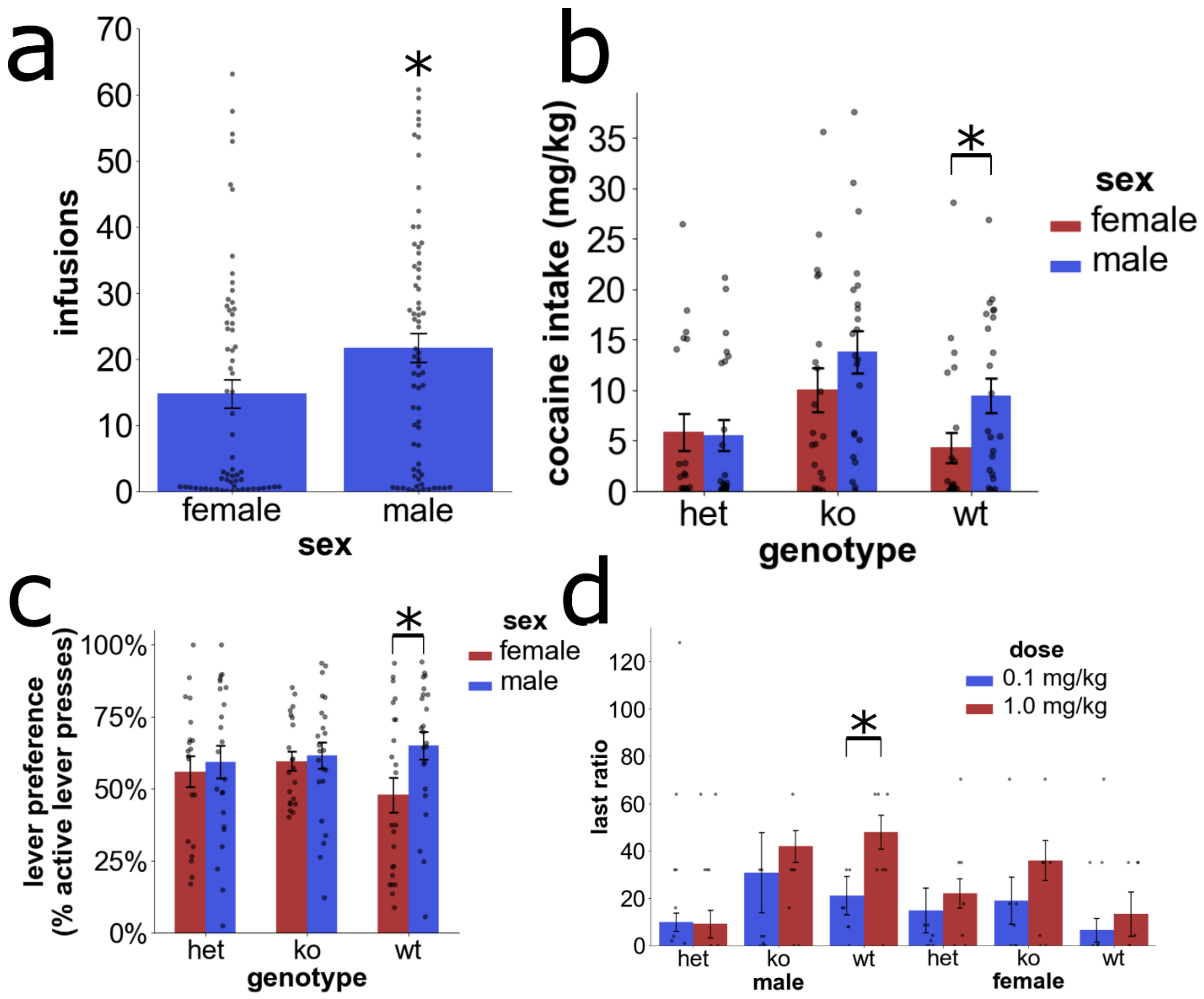

Supplementary Figure 5. Sex effects for cocaine self-administration (* = p<0.05). (**a**) There was a main effect of sex on the infusions earned across 10 CSA sessions, and male mice earned more infusions. Each dot is the average number of infusions earned by one mouse of over the 10 sessions. (**b**) A genotype by sex interaction for cocaine intake was driven by wildtype males consuming more cocaine than females. (**c**) A genotype by sex interaction for lever preference was driven by wildtype males displaying a higher preference relative to females. (**d**) A genotype by sex by dose interaction for last ratio achieved in progressive ratio testing was driven by wildtype males earning more of the 1 mg/kg dose relative to 0.1 mg/kg. The error bars show the SD in each of the panels.

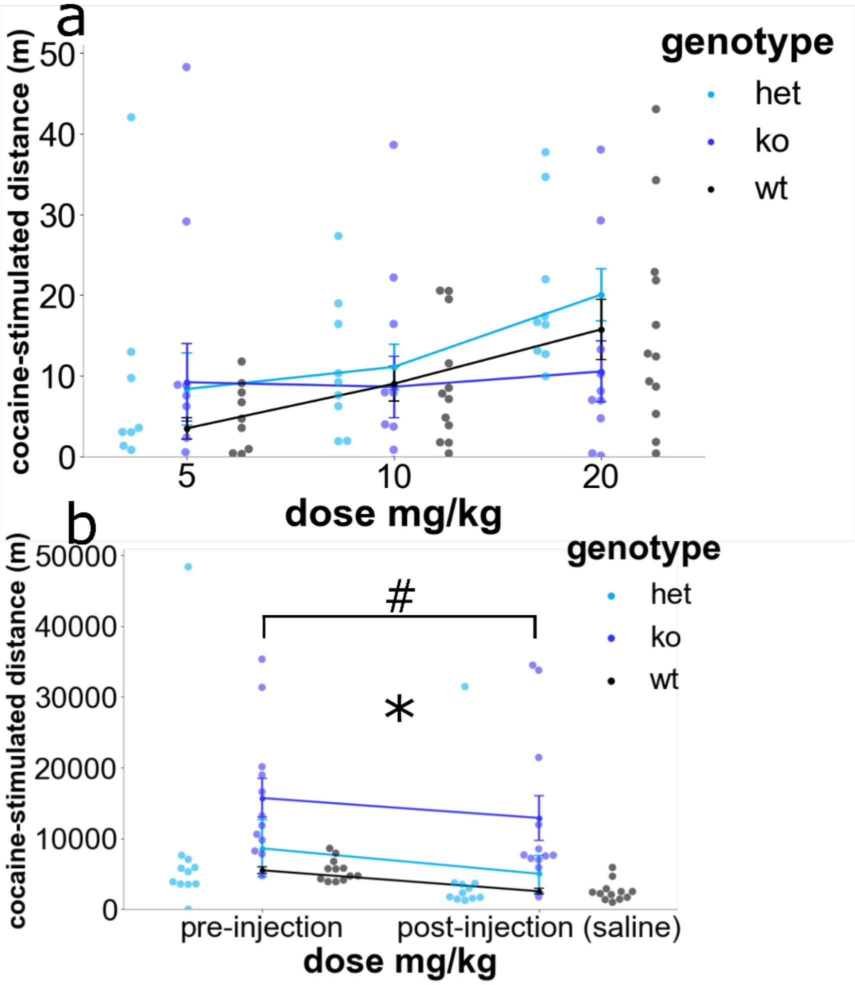

Supplementary Figure 6. The *Nav1* KO effect on the acute locomotor response to cocaine. (**a**) Acute locomotor response to cocaine injection in all 3 genotypes of the *Nav1* KO mice. The distance traveled differed across dose ($p<0.05$). However, no significant differences were detected between the mice with the three genotypes in either 30 min bins or when the data was combined ($p>0.05$) (the graph depicts the full hour). (**b**) To determine if there were basal differences in locomotor activity and potential genotype-saline injection interactions, we compared the distances traveled during the pre-injection period for the 1st saline session with the post-injection period (of the same time length). There was a main effect of genotype (* $p<0.05$); Nav1 KO mice traveled a greater distance than Wt and HET mice in both the pre-injection and post injection periods. We found that there was a main effect of injection (# $p<0.05$): mice of all genotypes traveled less in the post-injection period. The error bars show the SD in each of the panels.

a

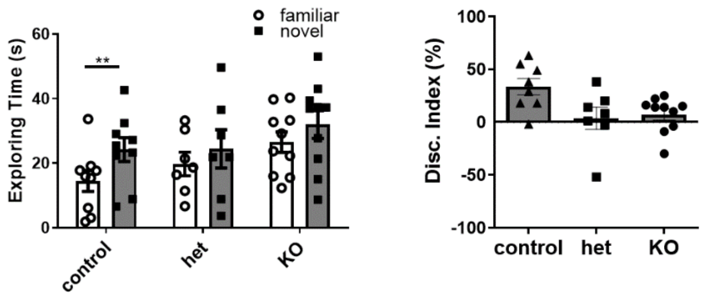

b

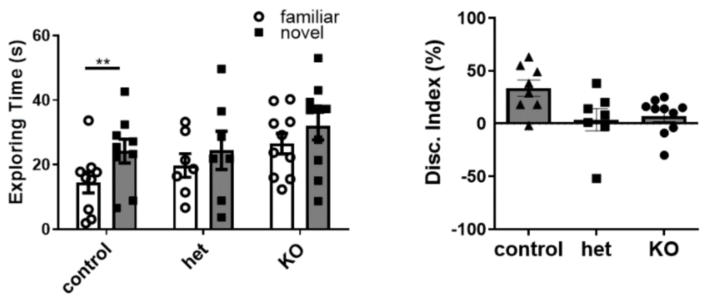

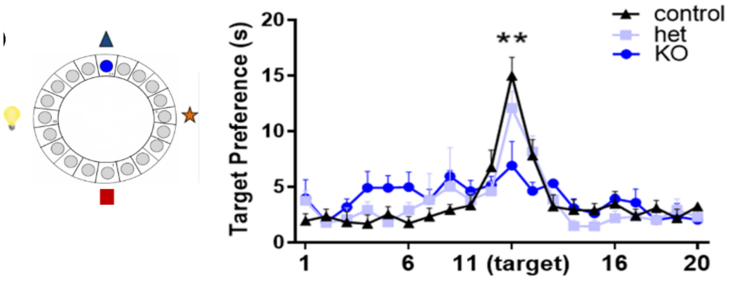

Supplementary Figure 7. Nav1 KO mice have disrupted novel object recognition and spatial learning abilities. **(a)** The novel object recognition test evaluates their preference for exploring a novel or familiar object. The left graph compares the time spent exploring a novel or familiar object (** , $p<0.01$). In the right graph, the discrimination index is calculated as the percentage of time spent with (novel – familiar object)/ total exploration time. Each bar represents the mean ± SEM for measurements made on 9 C57BL/6J (control), 7 HET, and 10 *Nav1* KO mice. Control mice spent more time with the novel object (**, $p<0.01$), while HET and homozygous *Nav1* KO mice did not. (**b**) The Barnes Maze test evaluates spatial learning and memory abilities. A schematic diagram indicates how the ability of a mouse to correctly identify a target escape hole (shown in blue) with the aid of visual cues that are shown outside the circle. The graph shows the time spent at the target hole ± SEM during a 90 second test session, which

was measured after 12 training sessions. The *Nav1* KO mice had a significantly reduced ability (** $p<0.01$) to correctly identify the target hole. Each data point is the average of measurements made on 6 control, 7 HET *Nav1*, and 4 homozygous *Nav1* KO mice.

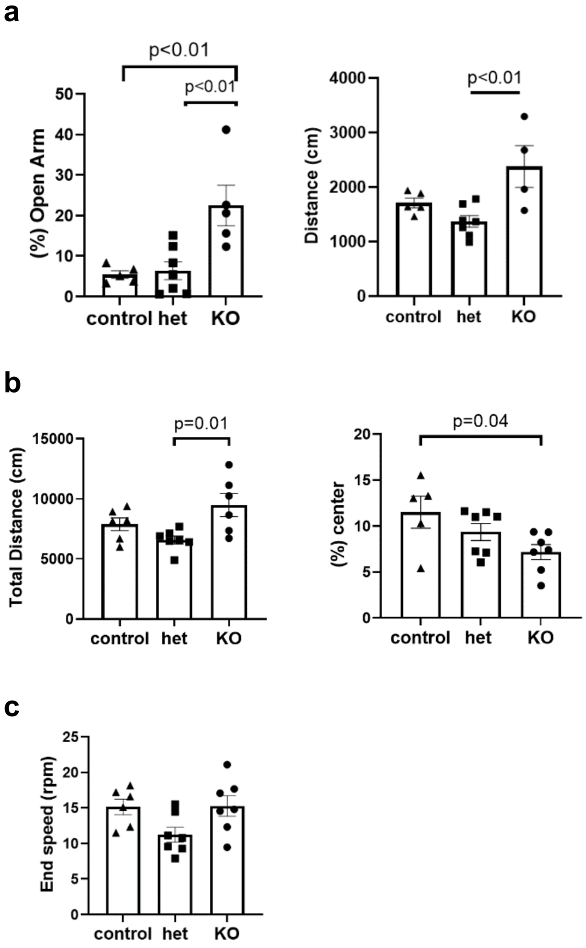

Supplementary Figure 8. *Nav1* KO mice have increased basal locomotory activity, altered exploratory behavior, and normal motor coordination. (**a**) The exploratory **(left)** and locomotor activities **(right)** of C57BL/6 (control), het Nav1 KO and homozygous *Nav1* KO mice were measured in the elevated plus maze test. (**b)** In the open field test**,** *Nav1* KO mice travel a greater distance but spend less time in the center than control mice. (**c**) In the rotorod test, the motor coordination of Nav1 KO mice is like that of control mice (p>0.99). In these graphs, the bars are the mean ± SEM of measurements made on 5 – 7 mice per group. The p-values where there were significant differences between groups of mice are indicated above the graphs.

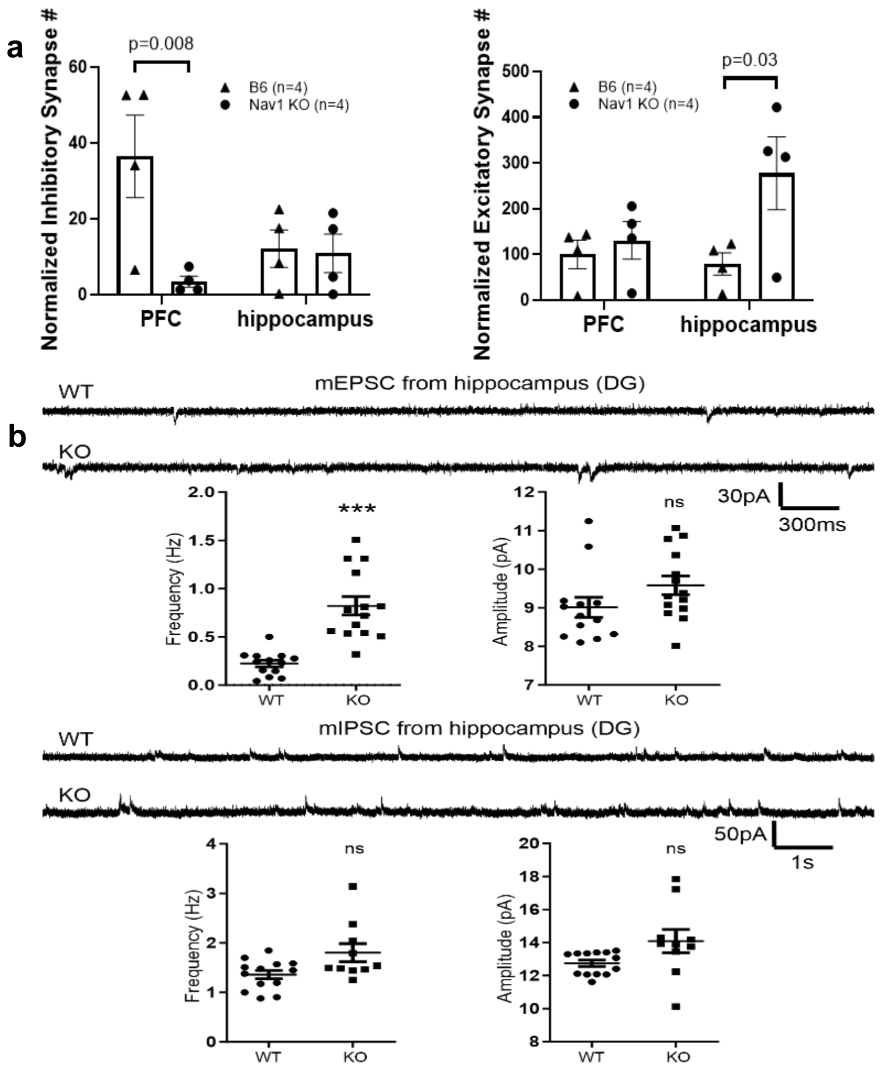

Supplementary Figure 9. The *Nav1* KO alters the excitatory and inhibitory balance in the PFC and hippocampus and increases excitatory synaptic transmission in the hippocampus. **(a)** Excitatory and inhibitory synapse formation in *Nav1* KO mice is disrupted. Brain slices prepared from hippocampal and PFC of *Nav1* KO mice were immunostained with antibodies to inhibitory presynaptic (vGat) and post-synaptic (gephyrin) marker proteins; or with antibodies to excitatory presynaptic (vGlut1) and post-synaptic (PSD95) marker proteins. Each bar is the average ± SEM of the number of inhibitory or excitatory synapses measured in PFC or hippocampal slices generated from four isogenic C57BL/6J or *Nav1* KO mice. Inhibitory synapse density in the PFC was significantly decreased (p=0.008) while the excitatory synapse density in the hippocampus was increased (p=0.03) in *Nav1* KO mice. (**b**) Excitatory synaptic

transmission in the dentate gyrus of the hippocampus of *Nav1* KO mice is increased. Representative traces of mEPSCs (Top) or mIPSCs (Bottom) recorded from granule cells in the dentate gyrus of the hippocampus of C57BL/6J (WT) and *Nav1* KO mice. The graphs show a significant increase in mEPSC frequency in granule cells of *Nav1* KO (vs. wildtype) mice, but not in their amplitude. In contrast, there was no significant difference in mIPSC frequency or amplitude between *Nav1* KO and WT mice. ***P < 0.001 by unpaired t-test. ns: not significant.

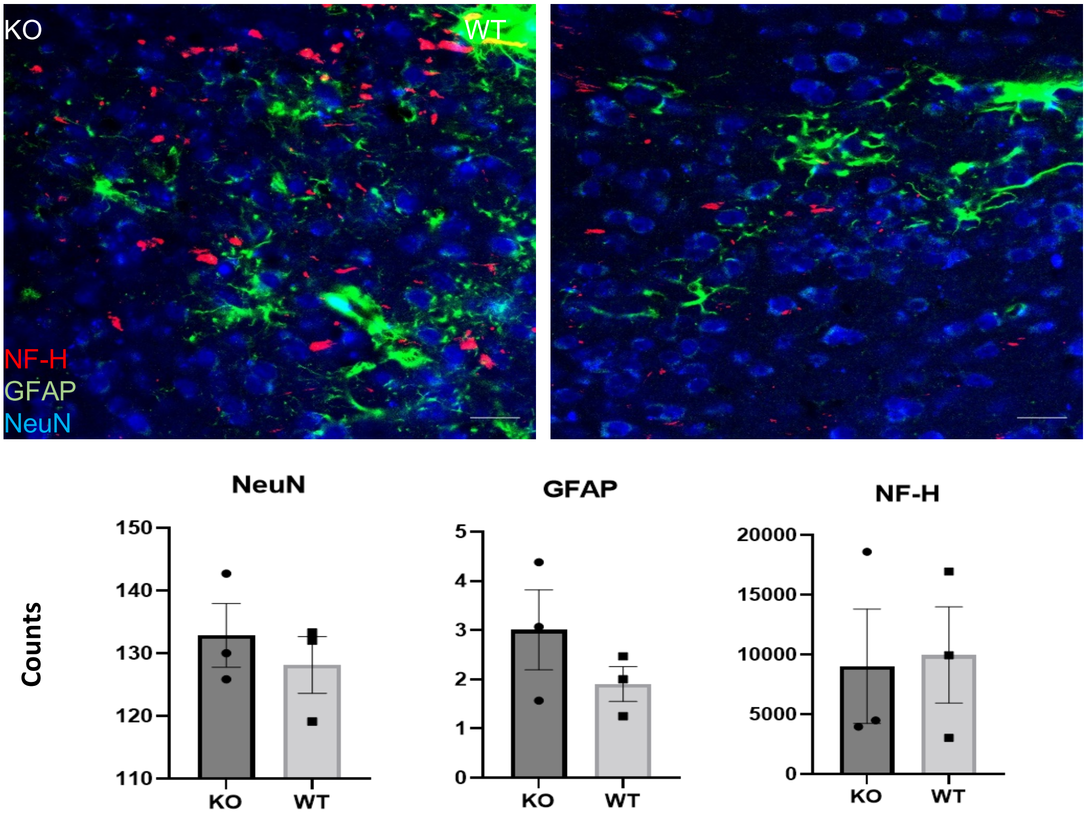

Supplementary Figure 10. The cells in the nucleus accumbens were not altered *in Nav1* KO mice. *Top*: Confocal images of representative sections of the nucleus accumbens that were obtained from *Nav1* KO (left) and C57BL/6 (right) mice that were stained with antibodies to neurofilament heavy chain (NF-H), glial fibrillary acidic protein (GFAP), and neuronal nuclear protein (NeuN). Scale bar: 20 um. *Bottom*: Immunostained sections of the nucleus accumbens were analyzed using CellProfiler software (the Broad Institute of MIT and Harvard) [3]. Each bar is the average $\pm$ SEM of analyses performed on 16 projection images (produced from 2.54 um z-stack) of 3 animals/group. There was no significant difference in the level of expression of NeuN (p value =0.53), GFAP (p value =0.28) or NF-H (p value =0.89) in the nucleus accumbens of *Nav1* KO (vs. C57BL/6 mice).

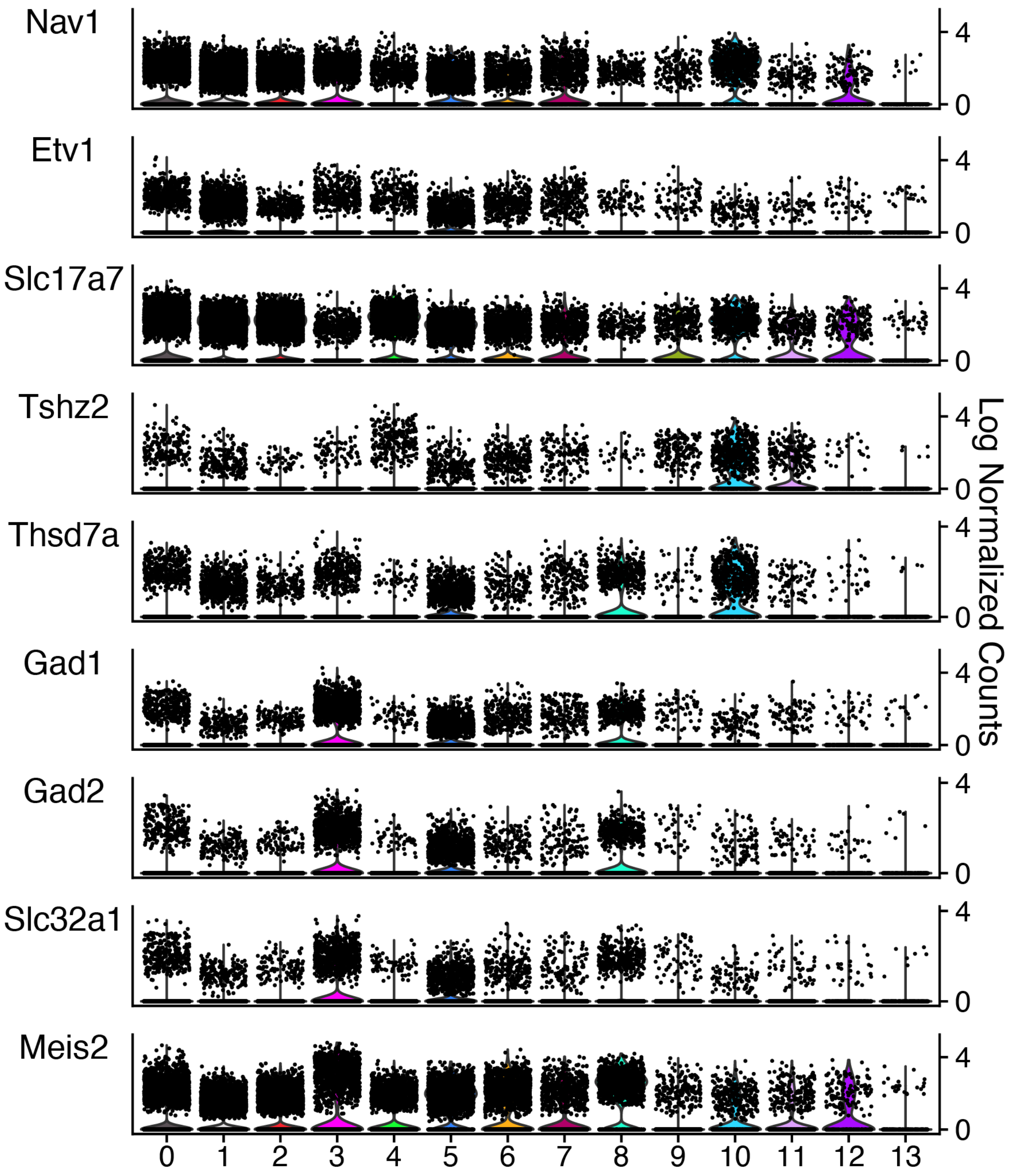

Supplementary Figure 11. Violin plots showing the level of expression of *Nav1*, *Etv1* and 7 neuronal marker mRNAs within 14 cell clusters identified in the PFC. The cluster number is indicated on the x-axis; and the y-axis shows the natural log transformed and normalized level of expression of each mRNA. Neuronal cluster identity was determined by expression of canonical mRNAs [4] for excitatory (*Slc17a7, Tshz2, Thsd7a*) and inhibitory (*Gad1, Gad2, Slc32a1, Meis2*) neurons. Cluster 10 was the only cluster with a

defined lineage that expressed a marker (*Tshz2)* for layer 5 cells, which are the corctical neuron whose transcriptome was altered during cocaine withdrawal [4]. Cluster 11 identity could not be determined due to expression of mRNAs for different lineages. Cluster 5 cells uniquely expressed mRNA for a transcription factor (*Etv1*) required for habitual behavior [5].

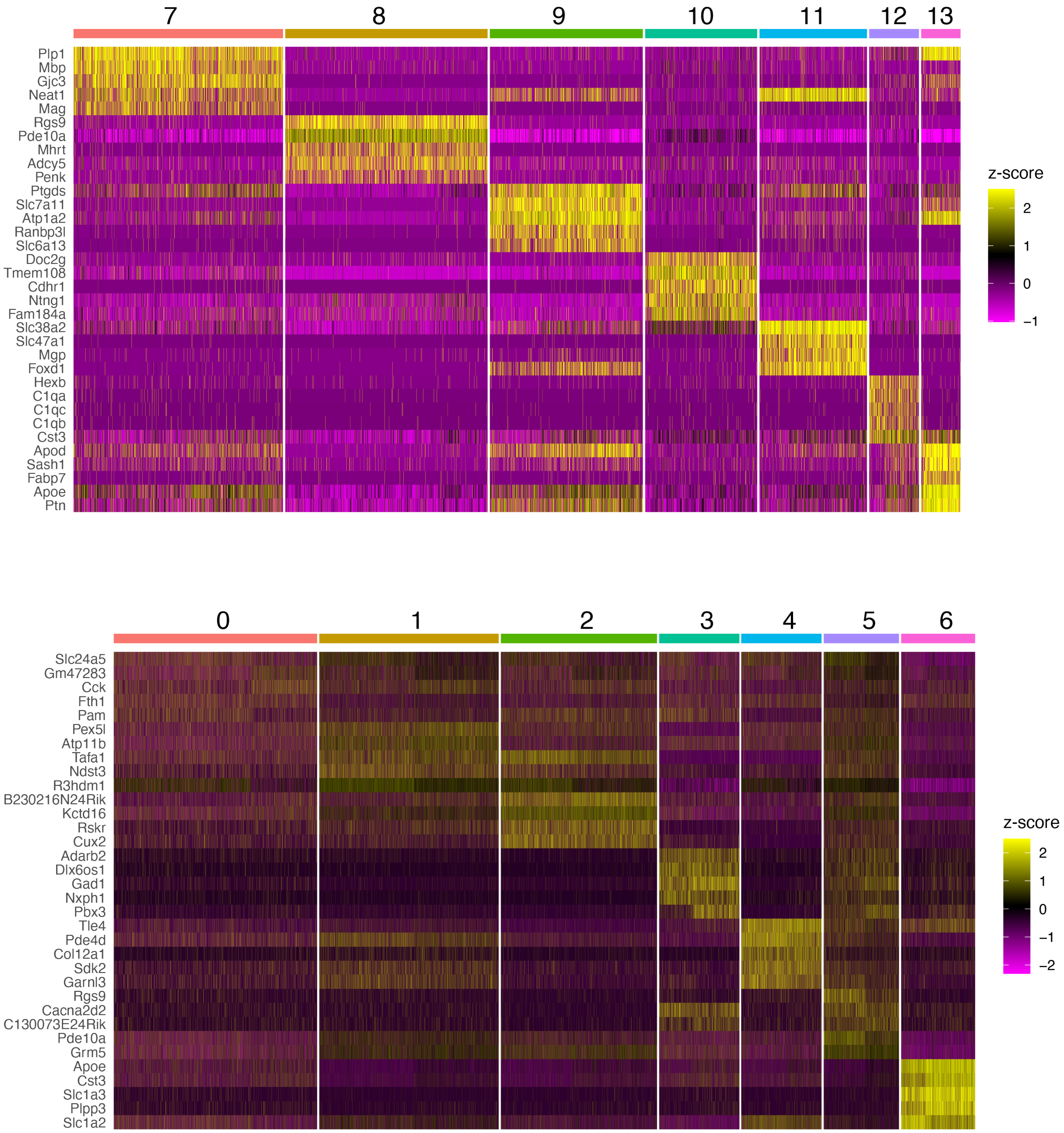

Supplementary Figure 12. A heat map showing the 5 most differentially expressed genes (DEG) for each of the 14 clusters identified in the analyzed PFC samples. The gene symbols are shown on the left. The expression level for each colored box in the heat map is shown on the right as the z-score of the log normalized expression counts. The DEGs have a mean $\log_2$(fold-change) of 2.2 and a mean percentage of 0.65 in that cluster.

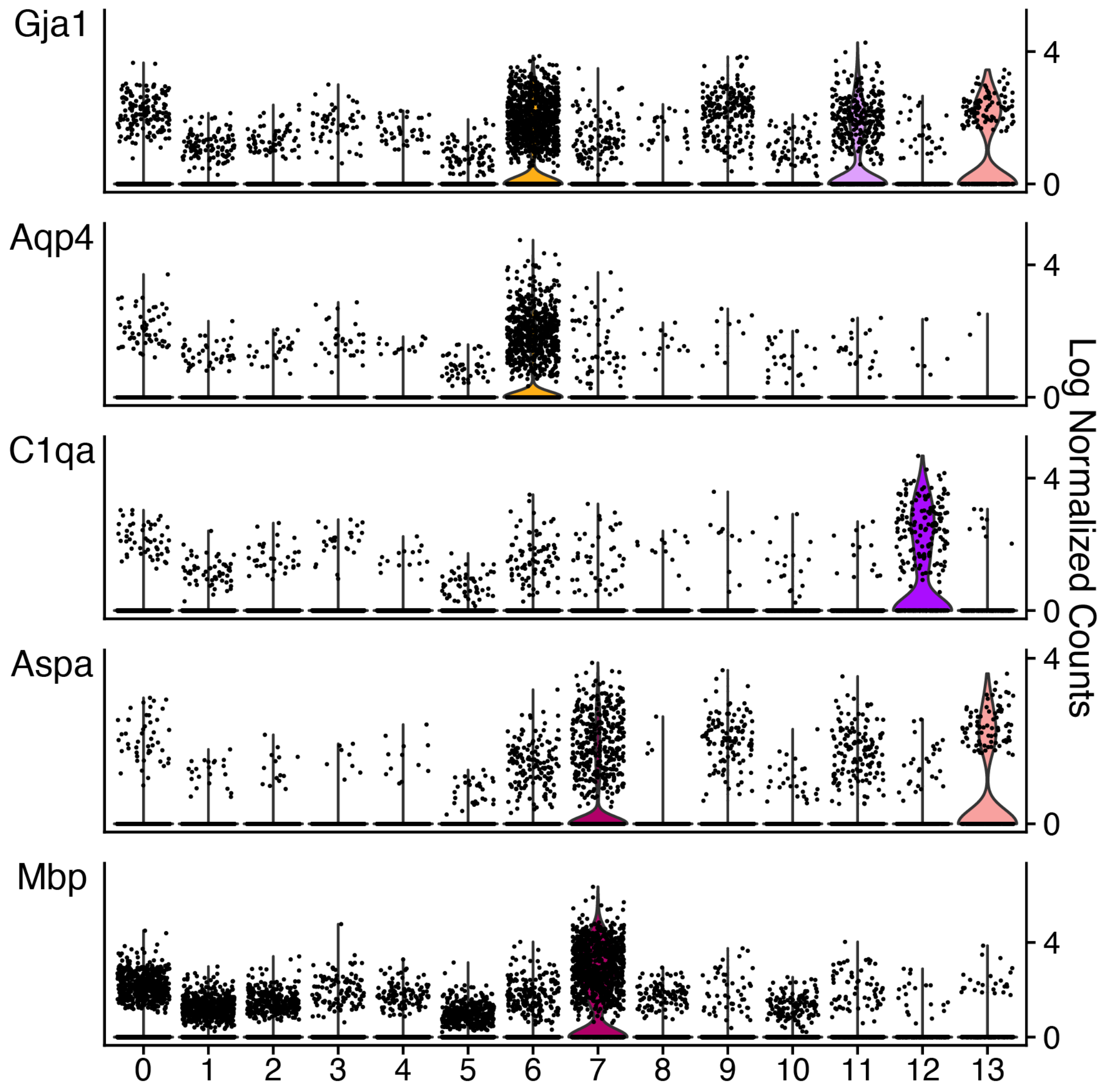

Cell Type	mRNA	Clusters
Astrocyte	*Gja1, Aqp4*	6, 13
Microglia	*C1qa*	12
Oligodendrocyte	*Aspa, Mbp*	7

Supplementary Figure 13. Genes whose mRNAs are specifically expressed in specific cell clusters in the PFC, which were identified by scRNA-Seq analysis. *Top*: Violin plots showing the level of expression of non-neuronal cell markers within each of the 14 cell clusters identified in the PFC. *Bottom:* The identity of the non-neuronal cell clusters was determined by their pattern of expression of the indicated canonical marker mRNAs [4]. The cluster number is indicated on the x-axis; and the y-axis shows the natural log transformed and normalized level of expression of the indicated mRNA in each cluster.

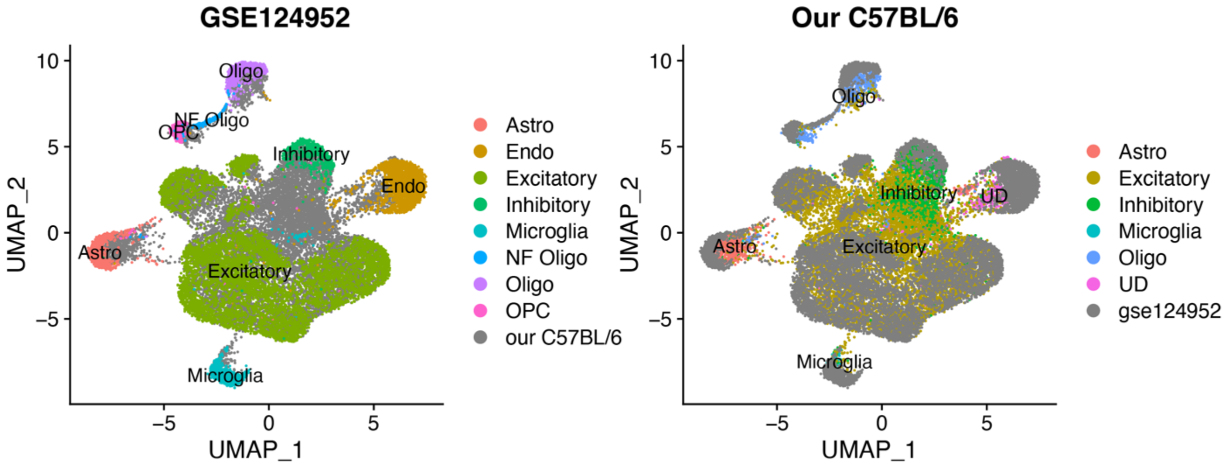

Supplementary Figure 14. UMAP plots comparing a published scRNA-Seq dataset (GSE124952) [4] generated from PFC prepared from adult C57BL/6 mice with our C57BL/6 PFC snRNA-Seq data. Each plot presents the overlap of the cells from the two datasets. The cell types within the clusters for each dataset are indicated by the dot color. For comparison purposes, a gray dot on the left is a cell from our C57BL/6 dataset and a gray dot on the right is from a cell in the published dataset. These comparisons demonstrate the very high level of concordance between the cell clusters and their distribution in these two data sets.

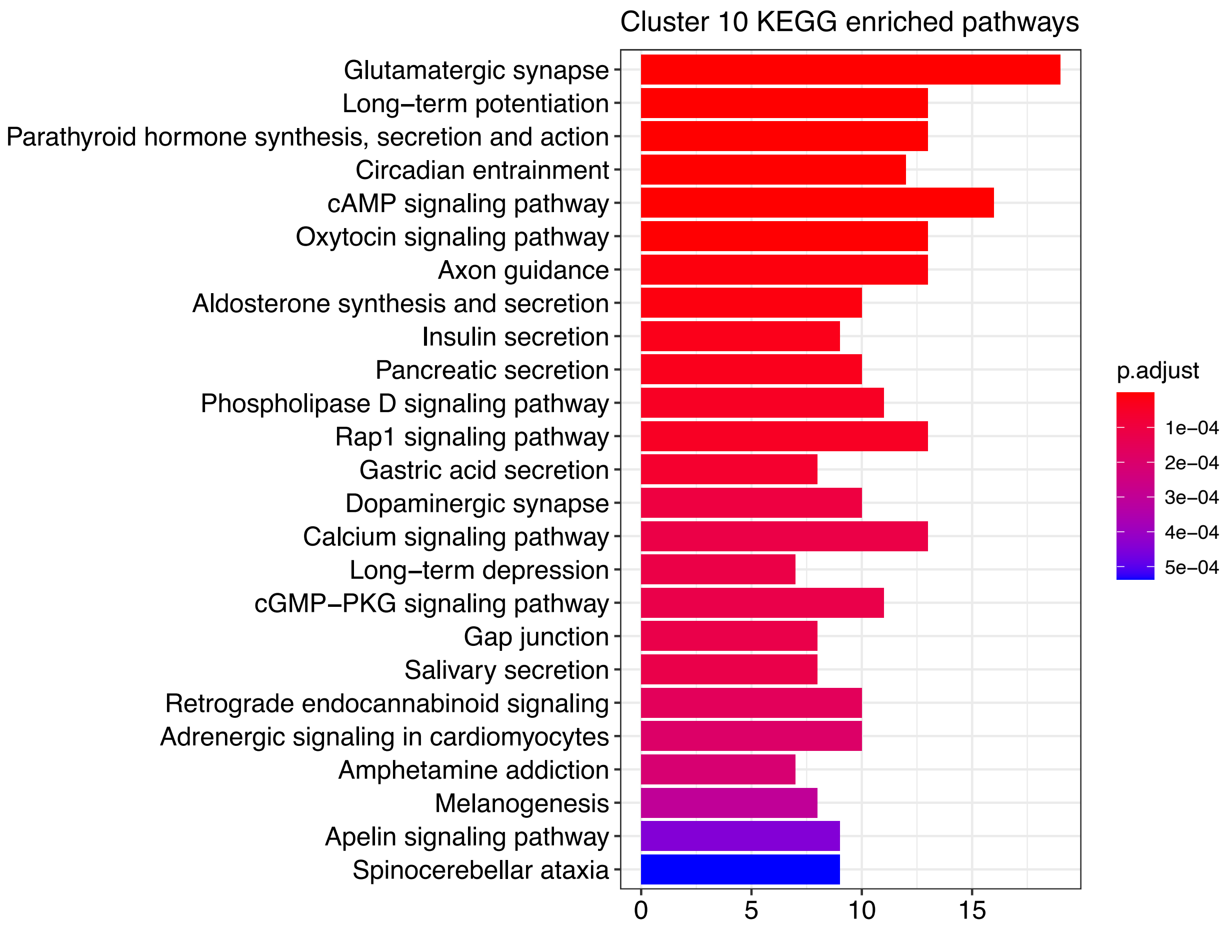

Supplementary Figure 15. This barplot shows the 25 KEGG pathways that were most significantly enriched when the DEGs in cluster 10 were analyzed relative to those in other excitatory neuronal clusters (0-2, 4, 5). The bar length indicates the number of DEGs within the indicated pathway. The FDR controlled p-values, which were calculated using the Benjamini Hochberg method, are indicated by their color (as shown on the right). While many of the enriched pathways were associated with neuronal guidance, synapse and signaling functions; seven of the DEGs in cluster 10 (*Gria3, Grin2b, Grin2a, Camk2a, Ppp3ca, Gria4, Prkcb*) were associated with an amphetamine addiction pathway (p=0.000025).

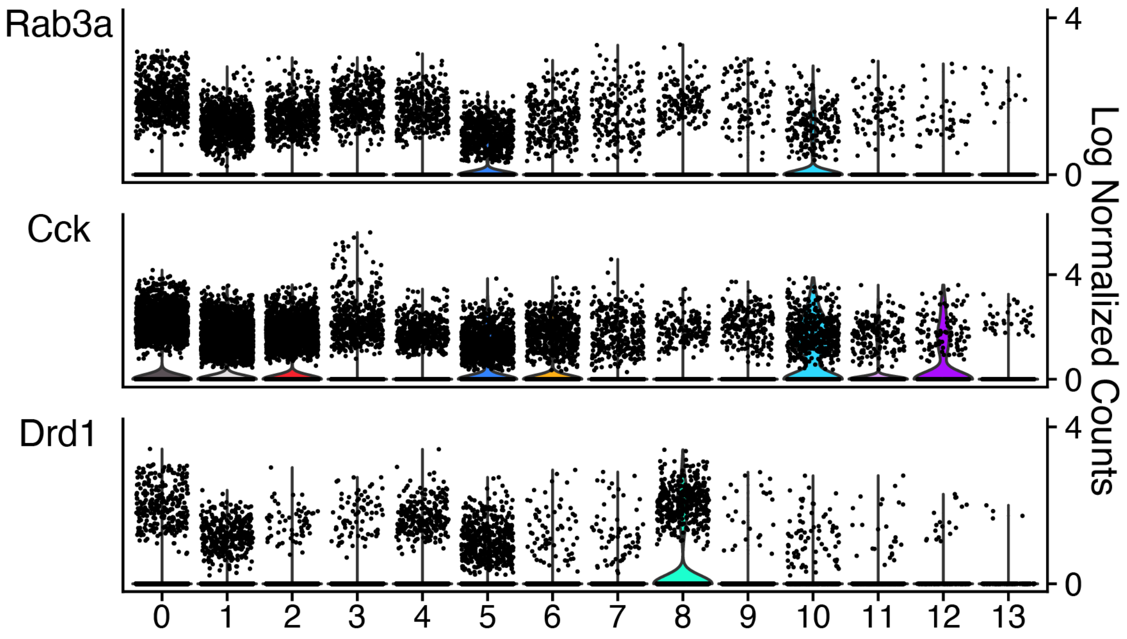

Supplementary Figure 16. Violin plots showing the level of expression of two mRNAs (*Rab3a* [6], *Cck* [7]) whose expression levels were shown to be altered during cocaine withdrawal [4]; and of *Drd1* mRNA, which was primarily expressed in cluster 8. The cluster number is indicated on the x-axis; and the y-axis shows the natural log transformed and normalized level of expression of the indicated mRNA in each cluster.
