# Supplementary material for: Neuron Navigator 1 (Nav1) regulates the response to cocaine in mice
Source: Commun Biol. 2023 Oct 18;6:1053. doi: 10.1038/s42003-023-05430-9 (PMC10584906; doi:10.1038/s42003-023-05430-9)
Supplement: Supplementary file 2 — Description of Supplementary Files [file 42003_2023_5430_MOESM2_ESM.docx]

**Description of Additional Supplementary Files**

**File name:** Supplementary Data 1

**Description:** The raw data used for preparing Figures 1A, 1D, 2 and 3 are contained in Supplementary Data File 1.
